# Single-cell and spatial transcriptomics define eosinophil heterogeneity during influenza A virus–induced exacerbation of fungal allergic asthma in C57BL/6 mice

**DOI:** 10.1093/jleuko/qiag120

**Published:** 2026-09-08

**Authors:** Anthony J Veltri, Anthony J Wells, Magdaline Baus, Sameer K Mathur, Amali E Samarasinghe

**Affiliations:** Biotechnology Center, University of Wisconsin-Madison, 425 Henry Mall, Madison, WI 53706, United States; Children's Foundation Research Center, University of Tennessee Health Science Center, 50 North Dunlap Street, Memphis, TN 38103, United States; Department of Pathology and Laboratory Medicine, TRIPath Core, 1111 Highland Avenue, Madison, WI 53705, United States; Department of Medicine, Division of Allergy Pulmonary and Critical Care, University of Wisconsin-Madison, 600 Highland Avenue, Madison, WI 53792, United States; Children's Foundation Research Center, University of Tennessee Health Science Center, 50 North Dunlap Street, Memphis, TN 38103, United States; Department of Medicine, Division of Allergy Pulmonary and Critical Care, University of Wisconsin-Madison, 600 Highland Avenue, Madison, WI 53792, United States

**Keywords:** Alox15, cell-cell interactions, eosinophil states, lung immunity, macrophages

## Abstract

Although eosinophils (EOS) are accepted as transcriptionally heterogeneous in asthma, how allergic inflammation shapes EOS states during influenza A virus (IAV) infection remains undefined. We integrated high-resolution single-cell RNA sequencing, spatial transcriptomics, quantitative immunofluorescence imaging, and cell-cell communication analyses to profile murine lungs across naïve, asthma, influenza (flu), and co-morbid asthma and flu (asthma + flu) conditions. We identified 3 related EOS transcriptional states distributed along a disease-associated continuum. EOS in the flu-only condition preferentially expressed canonical antiviral and interferon-stimulated genes, including *Isg15*, *Gbp2*, and *Samhd1*, whereas asthma + flu EOS had attenuated induction of these programs together with enrichment of genes associated with chemotaxis, adhesion, inflammatory regulation, and metabolic remodeling. Asthma + flu EOS were also enriched for a human asthma-associated high EOS gene signature. A progressive shift in EOS from antigen-presenting programs toward chemotactic and tissue remodeling-associated states, as identified by pseudotime analysis, may imply a changing functional emphasis rather than sustained canonical antiviral activation. Spatial profiling confirmed condition-dependent EOS abundance, increased EOS *Alox15* expression in allergic conditions, and disease-specific cellular neighborhoods. Cross-platform analyses identified recurrent predicted interactions between EOS and a distinct Alox15^+^ alveolar macrophage population. Mast cells adopted infection-specific interleukin-1 and leukocyte-activation transcriptional programs without major compositional changes. Collectively, these findings demonstrate that the type 2 microenvironment reshapes both the transcriptional state and spatial organization of lung EOS during IAV infection, providing an integrated framework for understanding EOS heterogeneity and cellular coordination in the allergic lungs.

## Teaser

Allergic asthma reshapes the transcriptional states and spatial organization of lung eosinophils during influenza A virus infection.

## 1. Introduction

Eosinophils (EOS) are multifunctional granulocytes that contribute to tissue remodeling, immune regulation, and host defense across diverse physiologic and pathologic contexts.^1–3^ This expanded view reflects a shift from the simplistic effector cell paradigm toward appreciation of EOS as dynamic, context-dependent cells with phenotypic and functional heterogeneity. Despite these conceptual advances identifying novel EOS functions, EOS have largely been treated as a single homogenous population in most mechanistic studies. Traditional approaches, such as the use of conventional surface markers for multiparameter flow cytometry and bulk transcriptomics,^4,5^ inherently average across cell states and obscure any phenotypic or functional heterogeneity that may exist in the EOS population. This technical limitation may have contributed to the enduring viewpoint that all EOS are created equally and that these uniform populations either drive pathology or support protective immune responses.

Early work suggested EOS heterogeneity at the tissue level, including distinct “tissue-resident” versus “inflammatory” subtypes based on activation state-associated markers.^6^ Resident EOS in the mouse lung are interleukin-5 (IL-5) independent and exhibit phenotypic and functional differences from inflammation-associated IL-5-dependent EOS recruited during type 2 (T2) allergic stimuli.^6^ Activation-dependent EOS phenotypes have been identified during allergen and/or influenza A virus (IAV) stimulation which also diverges based on IL-5 receptor expression.^7^ While these prior findings underscored that EOS subsets are context specific and presumably functionally divergent in health and disease, they did not provide a comprehensive transcriptional framework for EOS diversity in the lung mucosa.

Single-cell RNA sequencing (scRNA-seq) enables unbiased transcriptional profiling of individual cells and has been effectively used in varying mouse and human tissues to sub-classify cell populations and even identify new cell types.^4^ EOS have historically been underrepresented in rigorously performed scRNA-seq datasets,^8,9^ likely due to low EOS RNA yield, their intolerance to processing protocols, and high RNase content within granules—all of which contribute to technical challenges of capturing high-quality RNA transcripts from EOS. Through modifications and optimizations of scRNA-seq methodologies, multiple studies have now identified EOS in varying tissues at baseline and in varying disease states.^10–12^ In fact, Hu et al.^11^ presented an impressive profile of EOS across various tissues in naïve mice, whereas Goss et al.^13^ and Doni Jayavelu et al.^14^ reported subsets of EOS in patients with asthma. These recent datasets collectively suggest that, similar to other innate leukocytes, heterogeneity is a defining feature in EOS biology. However, gaps remain regarding EOS subpopulations across disease states in the lungs and in distinguishing true EOS intrinsic transcripts separate from contaminating lineages and artifacts. A rigorous, integrated transcriptional framework validated across multiple inflammatory settings is lacking.

Our group has studied the complex functions of EOS during asthma and influenza co-morbidity for the past decade.^7,15–17^ Our data hinted at heterogeneous populations of EOS in the lungs that are likely important in host defense, tissue remodeling and repair, and immune regulation.^7^ Following varying in-house testing of different platforms and methodologies, optimization, and cross-validation with our transcriptomic and proteomic datasets, herein we report on our use of high-resolution transcription profiling by scRNA-seq and spatial transcriptomics in the lungs from our mouse models of asthma, influenza, and co-morbidity and identify previously unrecognized EOS, macrophage, and mast cell (MC) transcriptional states.

## 2. Methods

### 2.1. Animals and ethical use of animals in research

Female C57BL/6 mice were purchased from The Jackson Laboratory (Bar Harbor, ME) at 6 weeks of age and acclimatized for 1 week in a temperature- and humidity-controlled room at the University of Tennessee Health Science Center (UTHSC) animal facility. Animals were placed in microisolator cages with autoclaved alpha-dri bedding and *ad libitum* access to autoclaved standard mouse chow (Teklad LM-485) and tap water. Cages were placed in ventilator racks connected to a Halcyon Air Tower. Animals were used in accordance with a protocol approved by the UTHSC Institutional Animal Care and Use Committee.

### 2.2. Asthma and influenza modeling

After 1 week of receipt, mice were enrolled in our allergic asthma and influenza co-morbidity model, described previously,^18^ with 3 animals allocated in each group for each experiment type. Briefly, mice were grouped as naïve, asthma-only (asthma), influenza-only (flu), and asthma and flu co-morbidity (asthma + flu). As shown in Fig. 1a, animals in the asthma and asthma + flu groups were subjected to *Aspergillus fumigatus* sensitization over a 5-week period, using whole-allergen extract from Greer Laboratories (Lenoir, NC). Anesthetized mice then were exposed to airborne conidia from an 8-day-old *A. fumigatus* culture for 10 min by inhalation. Two weeks later, the same allergen challenge by inhalation of fungal conidia was performed as previously described.^19–21^ Influenza A/CA/04/2009 original clinical isolate from the 2009 influenza pandemic was gifted by Dr. Richard Webby, St. Jude Children's Research Hospital. Virus was propagated in Madin-Darby canine kidney cells and sequence verified to be devoid of mutations from parental strain, and frozen in aliquots at −80 °C. Mice in the flu and asthma + flu groups were infected with 1,000 tissue culture infectious dose 50 of A/CA/04/2009 intranasally in a 50 µL suspension 7 d after the second fungal inhalation, as previously described^18^ and shown in Fig. 1a. Mice were weighed daily, starting before the virus infection and until the termination point, to determine loss of body weight, as is expected for IAV. In-depth characterization of this model has been published previously.^7,16–18,22^

**Figure 1:**
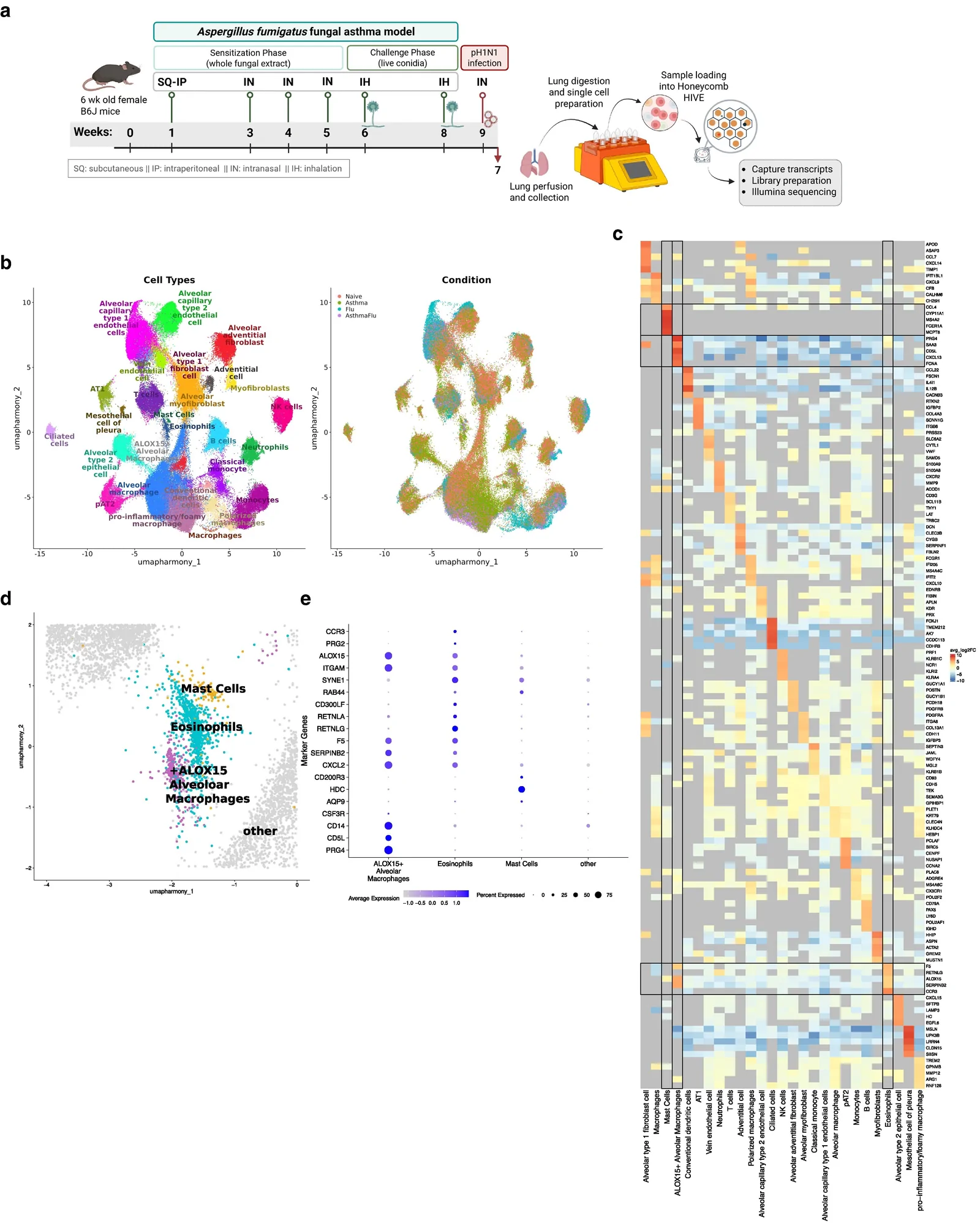
Single-cell analysis of lung tissue across conditions reveals distinct populations of MCs, EOS and macrophages. (a) Experimental overview of sample collection, infection timing, and single-cell library preparation (created in BioRender). (b) UMAP and clustering analysis with labeled cell types based on marker genes (left). UMAP analysis showing the condition of each cell in the embedding and visually demonstrating differences in abundance of cell types between disease conditions (right). (c) Heat map of log_2_-transformed fold changes of 5 top marker genes (adjusted *P* [p.adjust] < 0.05, by Wilcoxon RST; min_diff_percent >0.3) for each cell type. Gray boxes indicate that the given gene or cell-type pair did not meet criteria for inclusion in differential expression (DE) analysis (low fold-change/significance). (d) Zoomed-in view of cell types of interest representing EOS, MCs, and *Alox15*^+^ alveolar macrophages. (e) Marker genes comparing cell types highlighted in (d).

### 2.3. Sample collection and processing for scRNA-seq

At 7 AM on day 6 after the virus infection, mice in the naïve and asthma groups were euthanized per American Veterinary Medical Association guidelines, and lungs were perfused by 2 infusions, 10 mL each, of sterile PBS through the heart. Once lungs were perfused, they were excised and stored in PBS in ice. All processing steps were done on ice. Single-cell suspensions of the lung tissue were made with the GentleMACS mouse lung digestion kit (Miltenyi Biotec, San Jose, CA), and the 37 °C lung dissociation protocol on the dissociator was used. Red blood cells, if any, were lysed by mixing in 9 volumes of chilled sterile water followed by 1 volume of chilled 10 × PBS. We modified wash buffers by adding 25 µg/mL DNaseI (MilliporeSigma, St. Louis, MO) and 0.1 U/µL RNase inhibitor (MilliporeSigma). Cell suspensions were incubated in 1 µg/mL Brefeldin A (MilliporeSigma) for 30 min and filtered through 40 µm sieves. Cell counts and viability were measured.

Of the 3 samples processed per group, 2 samples per group were randomly selected for single-cell sequencing using the HIVE CLX scRNA-seq Capture, Processing, and Sequencing bundle platform from PerkinElmer (Hopkinton, MA) and Illumina sequencing (San Diego, CA). Following the manufacturer's protocol, HIVEs were thawed to room temperature and the storage buffer within the HIVEs was removed by pipetting. Lung cells from each sample were suspended in PBS/0.1% BSA at 30,000 cells/mL. From each sample, 1 mL of cells was added into independent HIVEs followed by 3 mL of PBS. The ports were covered and the HIVEs were placed at 4 °C for 15 min followed by a cold centrifugation at 30 ×*g* for 3 min. Media were pipetted out of the HIVEs and swirled once before removing the last 1 mL. Then 2 mL of sample wash buffer (provided with the HIVEs) was added into each HIVE and swirled gently. Wash buffer was removed from each HIVE by aspiration, and 2 mL of cell preservation solution was added and swirled gently to cover the HIVE surface. The ports were covered and HIVEs were placed flat in the −80 °C freezer until shipment.

The same process was repeated at 7 AM on day 7 after virus infection with the mice in the flu and asthma + flu group. Once all the HIVES were frozen, they were shipped overnight in dry ice to Revvity for library preparation, quality control (QC), and Illumina sequencing.

### 2.4. Sample processing for 10x Xenium spatial transcriptomics

Animals were taken through the asthma and influenza model as described (3 mice/group). After euthanasia and bronchoalveolar lavage, left lung lobes were excised and inflated with 10% normal buffered formalin. Samples inflated with formalin were embedded in paraffin (FFPE) and sectioned across the coronal place using nuclease-free techniques at a thickness of 5 µm and mounted onto 10x Genomics (Pleasanton, CA) Xenium slides for analysis. The samples were hybridized with the Xenium Prime 5K Mouse Pan Tissue and Pathways 10x Genomics Panel Set. Slides were processed following the 10x Genomics Protocol for Xenium In Situ Gene Expression. Cell segmentation staining was performed. Slides were imaged on the 10x Xenium In Situ Platform.

### 2.5. Sample processing for immunofluorescence

Following the Xenium experiment, consecutive lung FFPE tissue sections were cut at 5 µm. Tissues were deparaffinized and antigen retrieval was performed with steam in 1× Antigen Retrieval Buffer at pH 9.0 (Quanterix, Billerica, MA) for the first 2 cycles and at pH 6.0 (Quanterix) for the last cycle, followed by a 1-time, initial retrieval of Protease Plus (ACD, Newark, CA) for 30 min at 40 °C. Tissues were stained following the standard protocol from the Quanterix Opal 6-plex manual detection kit at room temperature as follows: CD68 (Abcam) at 1:50 for 30 min with Opal dye 520; 15-Lipoxygenase 1 (Abcam) at 1:300 for 15 min with Opal dye 650; and mEARs, a mouse EOS marker, (clone MT3-25.1.1, provided by Dr. Elizabeth Jacobsen at Mayo Clinic Scottsdale) at 1:100 for 1 h with Opal dye 620. Goat anti-rabbit and goat anti-rat secondary antibodies were used (Abcam) followed by counterstaining with DAPI prior to mounting with ProLong Gold Antifade Mountant (Invitrogen, Carlsbad, CA). The stained multiplex slides were scanned on the Vectra Automated Quantitative Pathology Imaging System (Quanterix) at 40 × objective and images were deconvoluted using InForm Software, version 2.4 (Quanterix).

### 2.6. Single-cell sequencing data analysis

Data initial QC and cell quality subsetting was performed by Revvity and saved as a Seurat object. Subsequent data analysis was performed using Seurat 5.2.1.^23^ Raw counts were normalized and 3,500 variable features were selected using default parameters. Data were scaled and principal component analysis was run on the scaled matrix of variable features. Uniform Manifold Approximation and Projection (UMAP) analysis was run on the top 30 principal components. Data were assessed for batch effects between conditions and samples, and samples were integrated using the Harmony algorithm,^24^ using a theta parameter of 4. UMAP and FindNeighbors were run based on the Harmony dimensionality reduction of the first 30 Harmony dimensions. Clusters were identified using Louvain with multilevel refinement (algorithm = 2) and a resolution of 0.6. Cell types were identified by a combination of projection from public datasets and manual annotation via marker genes (identified by FindMarkers with default parameters). Doublets were identified on a per-sample basis using scDblFinder.^25^ Doublets were simulated doublets based on these clusters using synthetic doublet rates corresponding to the rates published by Revvity based on the number of recovered cells (14% for asthma and naïve, 9% for flu, and 7% for asthma + flu samples). Cells with doublet scores exceeding the scDblFinder calculated thresholds were excluded from further analysis. The cluster containing identified EOS was further subclustered (after identifying 400 variable features and reintegrating with Harmony) using 12 Harmony dimensions, a k parameter of 30, and a resolution of 0.1. Subsets were further identified as *Alox15*^+^ alveolar macrophages, EOS, and MCs based on marker genes. Each of these populations was further subclustered based on a similar workflow (variable feature selection, integration, cluster identification). Gene names in figures are capitalized to improve legibility.

### 2.7. Immunofluorescence data processing

DAPI channels of immunofluorescence images for each field of view (FOV) were normalized using csbdeep (default clipping value parameters: pmin = 1, pmax = 99.8), segmented with StarDist,^26^ using the precomputed 2D_versatile_fluo model. Nuclear segmentations were expanded by 5 pixels using skimage.segmentation.expand_labels to capture additional cytoplasmic area. Mean fluorescence intensity for all channels was calculated for each cell segmentation using skimage. Threshold cutoffs were set manually to categorize each cell as mEARs-positive or ALOX15-positive based on histogram distributions of signal intensity. Centroid coordinates of each cell were also calculated by skimage.^27^ EOS (mEARs+ cell) percentages and average numbers per FOV were calculated on a per-FOV basis, then aggregated by sample and condition. Enrichment odds ratio calculation uses a Haldane-corrected odds ratio formula. For fields meeting a minimum cutoff of 50 cells, 15 EOS, and 15 non-EOS ALOX15^+^ cells, spatial co-occurrence was calculated between EOS and non-EOS ALOX15^+^ cells using squidpy.gr.co_occurrence^28^ on a per-field basis and averaged across each sample. The mean and SEM values of co-occurrence over all samples were then computed.

### 2.8. Xenium data analysis

Data from Xenium, including on-instrument cell segmentations, were loaded using the SpatialData^29^ and scanpy^30^ packages, and each sample was evaluated for quality. A minimum RNA count cutoff was applied at 28 counts/cell and cells with fewer counts were discarded for the purposes of transcriptomic analysis. Some samples, especially 15 naïve and 17 flu had a minority of their cells meeting this cutoff. Top variable genes from samples were identified, excluding 1 gene driving undesired cluster separation; then samples were integrated using Harmony, and a UMAP was generated using the top 10 components.

A classifier model was trained on shared genes from the single-cell dataset using CellTypist^31^ and used to predict cell types in Xenium on a per-cell basis. Marker genes were calculated based on >30% expression in the target cell type, and the top 5 genes for each cell type were plotted as a dot plot, excluding duplicates. EOS abundance percentage and *Alox15* positivity (defined as >0 *Alox15* counts) percentage was calculated as a percentage of cells meeting the QC cutoff. In addition to automated CellTypist identification, EOS were identified manually on the basis of aligned hematoxylin and eosin–stained images within selected regions across all tissues. These manual identifications included some cells filtered out by count-based QC. Spatial neighbor enrichment among predicted cell types was calculated for each Xenium section using an n_neighbors value of 6 (squidpy.gr.spatial_neighbors) and 1,000 permutations (squidpy.gr.nhood_enrichment). Cell types for which there were fewer than 10 cells labeled on a given section were excluded. Per-condition neighbor enrichment *z* scores were averaged. Significant enrichment was calculated using a 1-sample *t* test vs 0. Spatial cell-cell communication analysis was performed with LIANA+,^32^ using the liana.mt.rank_aggregate.by_sample method to calculate spatial cell-cell communication on a per-sample basis, with spatial proximity weighted by a Gaussian kernel with bandwidth of 250 µm. The “mouseconsensus” combined ligand-receptor database was used. Top ligand-receptor pathways were selected based on lowest average magnitude_rank (dot plots) or highest average lr_mean (heat maps). To enable comparison with single-cell data cell-cell communication results, the single-cell data were reanalyzed with LIANA+ using liana.mt.rank_aggregate per condition on the single-cell data. Top or shared pathways for comparison with Xenium analysis were chosen as the minimum mean magnitude_rank interaction for both datasets.

### 2.9. Statistical analysis

Statistical analyses for scRNA-seq between-condition comparisons were performed using the Model-based Analysis of Single-cell Transcriptomics (MAST)^33^ to account for biological variance between samples, using genes with a false discover rate of < 0.05. Comparisons to Doni Jayavelu et al.^14^ were completed using the top 50 genes for each of the high EOS (EosHigh) and low EOS (EosLow) signatures, and enrichment of these signatures were calculated using Seurat's AddModuleScore function. Compositional analysis was completed using the sccomp^34^ package. Pathway enrichment and pathway network analyses were performed using the Gene Ontology (GO) database and the clusterProfiler package.^35^ Pseudotime analysis was performed using Palantir.^36^ Cell-cell communication analysis was performed using CellChat^37^ independently for each condition and LIANA+ in aggregate (single cell) or spatially aware per slide (Xenium). *Alox15* expression differences among Xenium-predicted EOS was modeled using the statsmodels^38^ package for Python to fit *Alox15* log-normalized expression as an ordinary least squares linear model (alox15 ∼ condition + slide) and tested for condition effect by analysis of variance.

## 3. Results

### 3.1. scRNA-seq reveals a broad landscape of cell types in murine lung

To assay gene expression changes across the entire lung tissue in different conditions, we performed scRNA-seq using the Honeycomb HIVE platform in 2 mice each for 4 conditions: wild-type mice (naïve), the *A. fumigatus* fungal asthma model (asthma), pH1N1 influenza-infected mice 7 d after infection (flu), and fungal asthma model mice infected with pH1N1 7 d after infection (asthma + flu). Whole lung tissues from these mice were perfused and digested to create a single-cell suspension, which was subsequently prepared for single-cell barcoding and sequencing (Fig. 1a). In total, nearly 173,000 cells were detected across conditions, with recovery varying between conditions in the approximate range of 10,000 to 30,000 cells per sample (Figure S1a). After batch correction (integration) between conditions and visualization of the dataset via UMAP, the high number of cells recovered allowed us to perform clustering to recover a broad range of cell populations across the lung. To ensure high quality in subsequent analysis, we then assessed cell populations for mitochondrial RNA content (Figure S1b) and potential doublets, observing doublet rates that were consistent with the published rates for the Honeycomb HIVE technology (Figure S1c). We removed potential doublets, and we used marker gene analysis and a combination of automated and manual cell-type identification to assign labels to major clusters of cells (Fig. 1b, left, and c). Macrophages and other immune cell types made up a large portion of the observed cells, with cell numbers within a given population sometimes varying among conditions (Fig. 1b and Fig. S1d), providing an early indication that this dataset captures disease-state-specific functions or transcriptional profiles of lung cell populations.

To focus our subsequent analyses, we first turned our attention to a cluster of cells that contained a combination of markers for several cell types. Subclustering this group of cells revealed 3 distinct populations: the largest was composed of EOS, the next largest comprised a distinct alveolar macrophage population, and the final cluster comprised MCs (Fig. 1d). The EOS subcluster expressed elevated levels of canonical EOS marker genes, such as *Ccr3*, *Prg2*, and *Retnlg*,^39,40^ while also expressing other genes known to be expressed in EOS and other cell types, including the coagulation factors *F5* and *Serpinb2*,^41–43^ as well as myeloid markers like *Itgam* (also called *Cd11b*) (Fig. 1e). These EOS also showed high expression of the gene *Alox15*,^44^ a characteristic shared by the alveolar macrophage subcluster, which we identified based on its near-exclusive expression of macrophage markers like *Cd14* (Fig. 1e). Interestingly, although other clusters of alveolar macrophages are present in this dataset, this subcluster is the only macrophage population that expresses the known macrophage gene *Cd5l*^45^ (which codes for a secreted glycoprotein, apoptosis inhibitor of macrophage) and is also the only positive cluster for *Prg4* in the dataset. The transcriptomic commonalities of these cells to the EOS population raised the intriguing hypothesis that EOS-macrophage crosstalk may contribute to allergic host defense during influenza. Finally, MCs were identified by their high expression of *Hdc*, *Cd200r3*, and *Aqp9* (Fig. 1e).^14^ The remarkable similarities among these cell populations despite their distinct identities led us to explore their characteristics further in the context of our disease models.

### 3.2. EOS subclusters have distinct transcriptional signatures that correlate with disease states

To better elucidate the unique characteristics of the lung EOS population, we then isolated these cells and reanalyzed them to produce a new UMAP representation and subclusters. We clustered cells into 3 groups with distinct transcriptional states. Despite batch correction between samples for visualization and clustering, we observed that the asthma + flu EOS formed a distinct cluster (Fig. 2a) and made up the majority of cluster 2, whereas cells from asthma and naïve samples were distributed among clusters 0 and 1. Cells from the flu group were also present in limited numbers throughout all 3 clusters. We then used cell-level differential expression analysis using the Wilcoxon rank sum test (RST) to identify genes that were more highly expressed in each cluster than all others.

**Figure 2:**
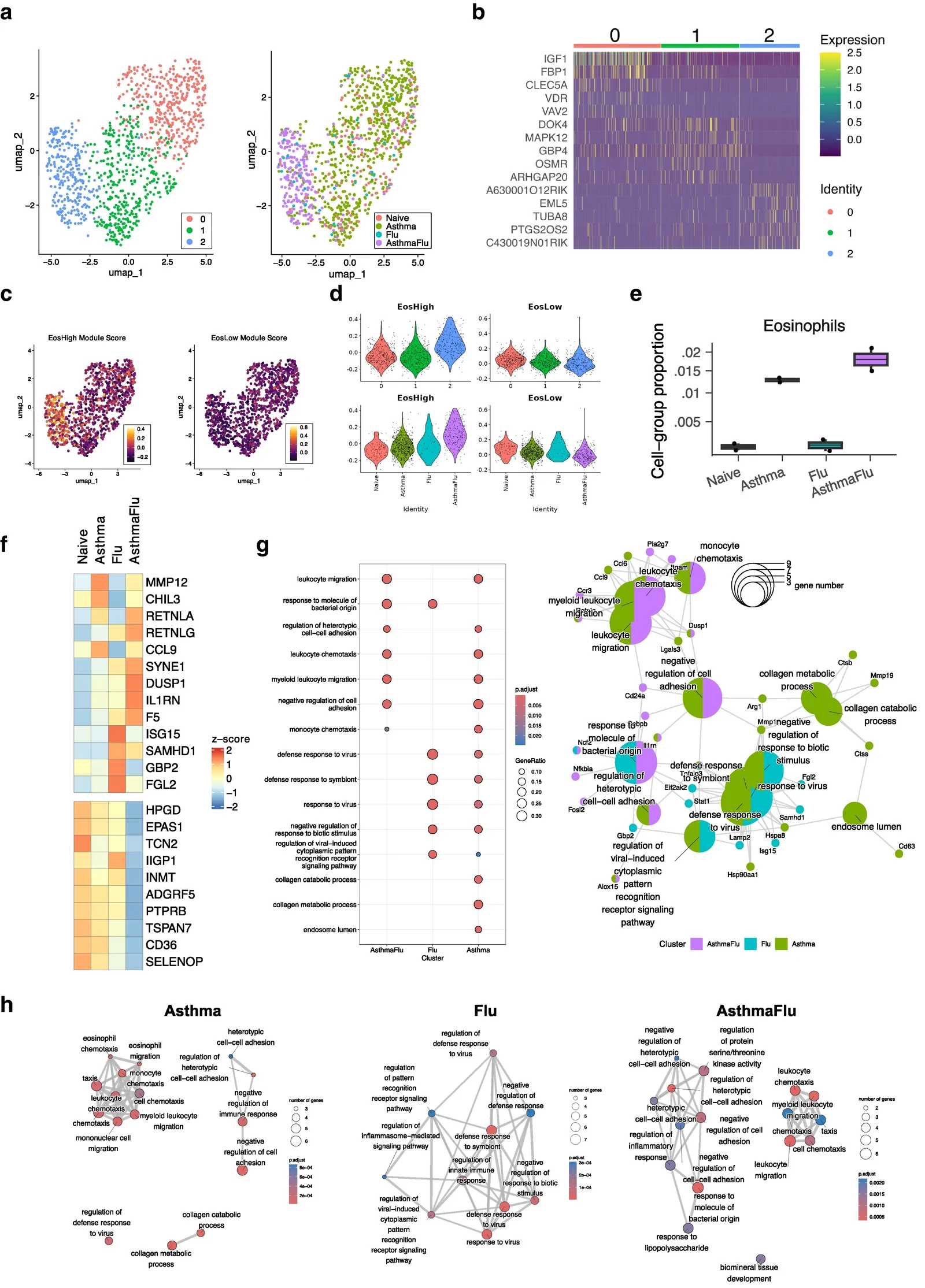
Analysis of EOS across conditions and comparison with known gene signatures. (a) UMAP reanalysis and re-clustering (left) of EOS after subsetting; condition represented as color for each EOS cell (right). (b) Top marker genes for each cluster are represented as a heat map, with each cell representing a column and each row representing a gene. Expression is plotted as log-normalized abundance. (c) ModuleScores for gene sets from Doni Jayavelu et al.,^14^ including EosHigh and EosLow signatures in human asthma. Higher numbers indicate more enrichment against a background set of genes. (d) EosHigh and EosLow ModuleScores plotted in each cell cluster (left) and in each condition (right) among EOS. (e) Compositional analysis plotting the EOS proportion relative to all cells for each sample. (f) Heat map of average expression of top MAST differentially expressed genes for each condition vs naïve (top panel) and bottom downregulated genes vs naïve (bottom panel). (g) Comparisons of major pathways in GO enrichment analysis (left). Network analysis (cnetplot) showing connections between major pathways between conditions (right). (h) Network analysis (emapplot) showing connections between enriched pathways in asthma, flu, and asthma + flu (each vs naïve) comparisons.

We identified genes including *Igf1*, *Fbp1*, and *Clec5a* as markers of cluster 0, with a gradual decrease in expression of these genes moving into cluster 1 (Fig. 2b). In cluster 1, genes *Dok4*, *Gbp4*, and *Osmr* increased in expression while still retaining some expression of the cluster 0 genes. In cluster 2, genes including *Eml5*, *Tuba8*, and *Ptgs2os2* were upregulated compared with the other EOS clusters. The pattern of gene expression indicates the possibility of a spectrum within the EOS transcriptome, where cells may express differing amounts of cluster 0 and cluster 1 markers, as do most asthma and naïve cells; and cluster 2, dominated by flu and asthma + flu cells (Fig. 2a), represents a further transition on this spectrum.

A recent study used RNA-seq to define EOS-specific gene signatures in children with asthma stratified by high and low EOS counts.^14^ Therefore, we asked whether these human EOS gene signatures were conserved in our mouse dataset. We converted the EOS gene signatures from patients with high and low EOS counts to the mouse gene equivalents and designated them EosHigh and EosLow, respectively. We then used a module score calculation that measured enrichment of the gene signature over a random background to assess the enrichment of these genes among our EOS subclusters (Fig. 2c). Although the EosLow signature remained relatively low across all EOS subclusters in our dataset, we observed a strong increase in the module score in our cluster 2 (Fig. 2c and d, left). Because this cluster was dominated by asthma + flu EOS, we next evaluated the module scores separating cells by their condition and observed that the asthma + flu samples, indeed, had generally elevated levels of the EosHigh signature (Fig. 2d, right). The EosLow signature remained relatively low among all conditions, indicating that this signature is largely absent from cells among our disease groups. This analysis provides evidence that similar gene expression patterns activated in response to asthma in patients with EOSHigh are present in allergic mice during exacerbation caused by IAV.

### 3.3. Disease response alters EOS number and gene expression pathways

Given that Doni Jayavelu et al.^14^ observed variations in the number of nasal lavage EOS among different classes of patients with asthma, and our past data indicated differences in EOS numbers in the bronchoalveolar lavage in this mouse model by flow cytometry,^18^ we questioned whether such changes were quantifiable in the scRNA-seq data among the disease conditions in the lungs. Although total recovered cell numbers varied among samples and were systematically different for disease conditions, true changes in relative proportions of cells could only be revealed by differential abundance or compositional analysis. We used the R package sccomp^34^ to account for these variations and test differences between EOS numbers in each disease state vs the naïve samples (Fig. 2e).

Despite low replicate numbers, we observed statistically significant elevations of EOS proportions in all 3 disease conditions, with the most dramatic increase observed in asthma + flu samples, which also had the most elevated EosHigh module score. Interestingly, despite the relative sparsity of EOS in the flu group in our dataset, they also were statistically significantly elevated above naïve levels when analyzed compositionally. Although we found a small amount of EOS in the BAL in the flu-control group by flow cytometry,^7,16,18^ their levels did not always reach statistical significance when compared with EOS in the naïve control,^18^ highlighting a limitation of surface marker staining and detection by flow cytometry.

We next aimed to find transcriptomic differences between each of our disease conditions. The cell-level differential expression performed to find marker genes between EOS clusters, as in Fig. 2b, performs well for finding cluster-specific genes; however, it can also be affected by inflated *P* values and false-positive results.^46^ To test condition effects, we can leverage more advanced statistical tools, including Hurdle models and random effects, to identify the genes most robustly changing between conditions. Therefore, we next performed differential expression analysis using the package MAST to compare each disease condition with the naïve state. The top 5 significantly upregulated and bottom 5 significantly downregulated genes for each comparison were plotted as a heat map, revealing patterns of gene expression unique to each condition (Fig. 2f). Both asthma conditions had relatively elevated levels of *Mmp12*, *Chil3*, and *Retnla*. Asthma + flu also had distinct upregulation of *Syne1*, *Dusp1*, *Il1rn*, and *F5*, a characteristic shared to a lesser degree by the flu condition. The flu condition additionally had robust upregulation of the antiviral and immune-related genes *Isg15*, *Samhd1*, *Gbp2,* and *Fgl2*, consistent with a response to flu infection. Interestingly, the asthma + flu condition did not have similar upregulation of *Isg15* and *Gbp2*.

Among genes downregulated in each condition (ie upregulated in the naïve condition), all were most downregulated in the asthma + flu condition, indicating broad perturbation of transcriptional programs in that condition. Interestingly, one gene related to antiviral response, *Iigp1*, actually was upregulated in flu vs naïve, yet was downregulated in asthma + flu vs naïve (Fig. 2f). These results point to a changing role of EOS in the asthma + flu dual-disease condition compared with asthma or flu alone.

To understand the changes in gene expression in a broader cellular context, we performed pathway enrichment analysis and biological theme comparison using the GO database^47,48^ for the top 25 most upregulated genes in each of the disease condition vs naïve comparisons. The asthma condition had broad overlaps with both the flu and asthma + flu conditions, whereas flu and asthma + flu had limited overlapping top pathways with each other (Fig. 2g). The flu condition tended to have strong signatures for viral response, with 4 of the 6 top pathways directly related to viral response. Meanwhile, asthma + flu had just one top pathway related to infection response (response to molecules of bacterial origin) and, instead, predominantly had top pathways related to migration and chemotaxis of leukocytes. Although the asthma condition also had some pathways related to viral response and defense, the upregulated genes that match these pathways (*Mmp12*, *Hspa8*, *Hsp90aa1*, and *Tnfaip3*) can be considered broad stress-response genes that are not highly specific for viral response, in contrast to some of the genes upregulated by flu, like *Stat1* and *Isg15* (Fig. 2g, right). Clustering these pathways with their top genes into a network diagram revealed patterns of interconnection between asthma and asthma + flu (driven by genes such as *Retnlg*, *Dusp1*, *Lgals3*, *Alox15*, and *Itgam*) and between asthma and flu (driven by genes such as *Mmp12*, *Fgl2*, *Hspa8*, and *Isg15*), whereas flu and asthma + flu had only limited overlap driven by genes like *Ncf2*, *Fosl2*, and *Cebpb* (Fig. 2g).

We constructed similar network diagrams for each of the top pathways within the individual disease groups and found interesting patterns. In the asthma condition, pathways generally clustered into two densely connected groups: those relating to adhesion and chemotaxis (Fig. 2h). The flu pathways generally were quite interconnected, though some modules were related to viral pattern recognition, viral response, and immune cell activation. Similar to asthma alone, asthma + flu showed two distinct modules: one group related to cell migration and chemotaxis, and another related to metabolic, developmental pathways, and cell adhesion. Notably absent were strong pathways related to the antiviral response.

### 3.4. Pseudotime analysis of EOS reveals distinct gene clusters along disease trajectories

Given the stepwise progression of gene expression along the EOS cluster marker genes (Fig. 2b), a possible interpretation is that the observed transcriptional states of EOS in our dataset represents a continuum with EOS, from naïve mouse lungs in their normal state representing one end of the spectrum to highly perturbed EOS from the asthma + flu mouse lungs representing the other end. To reveal potential patterns of gene expression along this disease progression, we ran an embedding of the cells as a force-directed graph using the top three diffusion components and performed pseudotime analysis using naïve-group EOS as the starting point (Fig. 3a and b). Indeed, the distribution of EOS from each group in this embedding corresponded with the idea that subsets of asthma + flu and asthma EOS had the highest pseudotime (transcriptomic distance) from the starting EOS in the naïve condition (Fig. 3b). Using imputation of gene expression via the Markov affinity-based graph imputation of cells (MAGIC) algorithm,^49^ smoothed gene expression trends can be calculated along this pseudotime and clustered into broad categories based on changes in their abundance over this trajectory (Fig. 3c). We chose a clustering resolution that revealed three trends: increasing (trend 1), increasing then decreasing (trend 2), and decreasing (trend 3).

**Figure 3:**
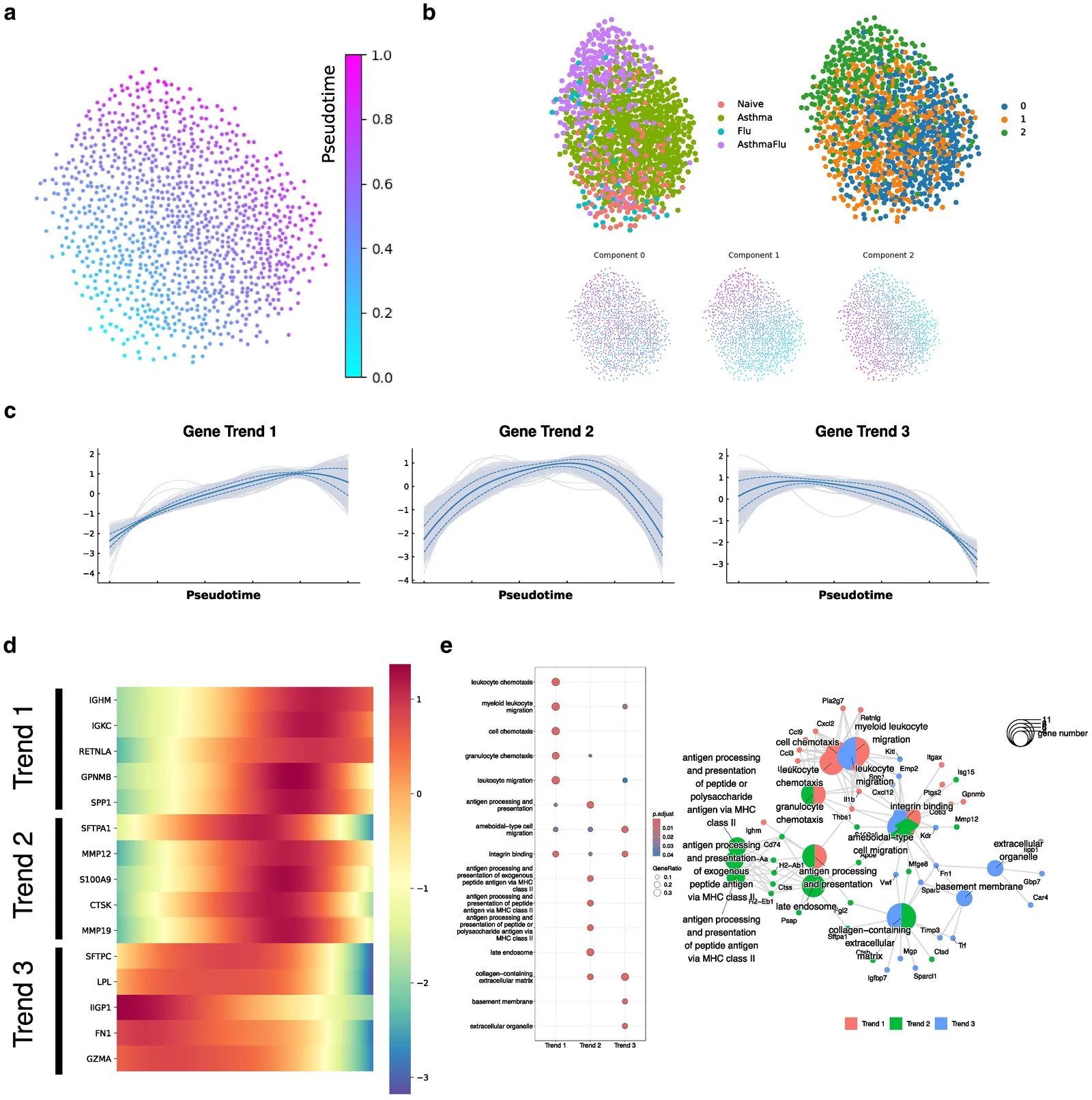
Pseudotime analysis of EOS populations and gene trends along pseudotime. (a) Force-directed graph embedding of EOS transcriptomes, colored by pseudotime. (b) Cells colored by condition, cluster from Fig. 2a, and major diffusion components. (c) Summary plots of MAGIC-imputed gene expression for genes associated with each gene trend: increasing (trend 1), increasing then decreasing (trend 2), and decreasing (trend 3). (d) Heat maps of MAGIC-imputed gene expression for each gene. (e) Comparisons of top pathways associated with each gene trend cluster. MHC, major histocompatibility complex.

We used standardized variances of gene expression across the EOS to find top genes from each of these trend clusters (Fig. 3d). Pathway enrichment analysis on the top 25 genes by standardized variance revealed some distinct biological functions related to the genes in each trend (Fig. 3e). Gene trend 1, which was increasing over pseudotime, had many pathways related to chemotaxis and migration, which agrees with the upregulation of these genes in the asthma and asthma + flu samples with higher pseudotime values. It additionally exhibited some pathways related to fatty acid metabolism and heat or fever generation (Figure S2a, left), consistent with a trend toward disease response in the asthma + flu samples. Trend 2 primarily included pathways related to antigen processing and presentation, indicating an immune role that becomes activated but may be downregulated in high-pseudotime cells among the asthma and asthma + flu samples. Previously, we showed that EOS played an antigen-presenting role including immune synapse formation during IAV infection to enhance CD8^+^ T-cell responses in the lungs.^17^ These data suggest that EOS subclusters in the lung may have been responsible for these immunoregulatory functions during influenza. Additionally, other pathways were detected relating to cholesterol biosynthesis and proteolytic activity (Figure S2a, middle). Trend 3 consisted of a decrease in expression and included some pathways related to migration, the basement membrane, and extracellular organelles, potentially indicating changes in EOS interactions with their microenvironment along the disease trajectory. Pathways such as cell killing and regulation of viral life cycle were also present (Figure S2a, right), potentially indicating a phenotype like exhaustion or tempering of the immune response among late-pseudotime EOS.

The EosHigh and EosLow module scores were also plotted against pseudotime (Figure S2b). EosLow did not appear to change along pseudotime; however, the presence of cluster 2 cells along the higher end of the pseudotime range caused the EosHigh score to be increased among this higher pseudotime range.

### 3.5. MCs take on divergent transcriptional programs based on disease state

The next cell type we investigated further was MCs. When reclustered and visualized via UMAP, two clearly distinct groups of cells emerged: one primarily with IAV infection (flu and asthma + flu conditions), and another comprising mostly cells of the naïve or asthma conditions (Fig. 4a). To identify genes that may be differentially expressed in these MC groups, we used the cell-level Wilcoxon RST to identify marker genes for each of the two clusters. Although cluster 0 markers, such as *Hpgd*, *Icam2*, *Vwf*, *Cdh5*, and *Inmt*, were expressed nearly exclusively in this cluster, it also shared some expression of cluster 1 markers, like *Cd7*, *Rgs1*, *Csrnp1*, *Slc18a2*, and *Igf1r*, albeit at lower levels (Fig. 4b).

**Figure 4:**
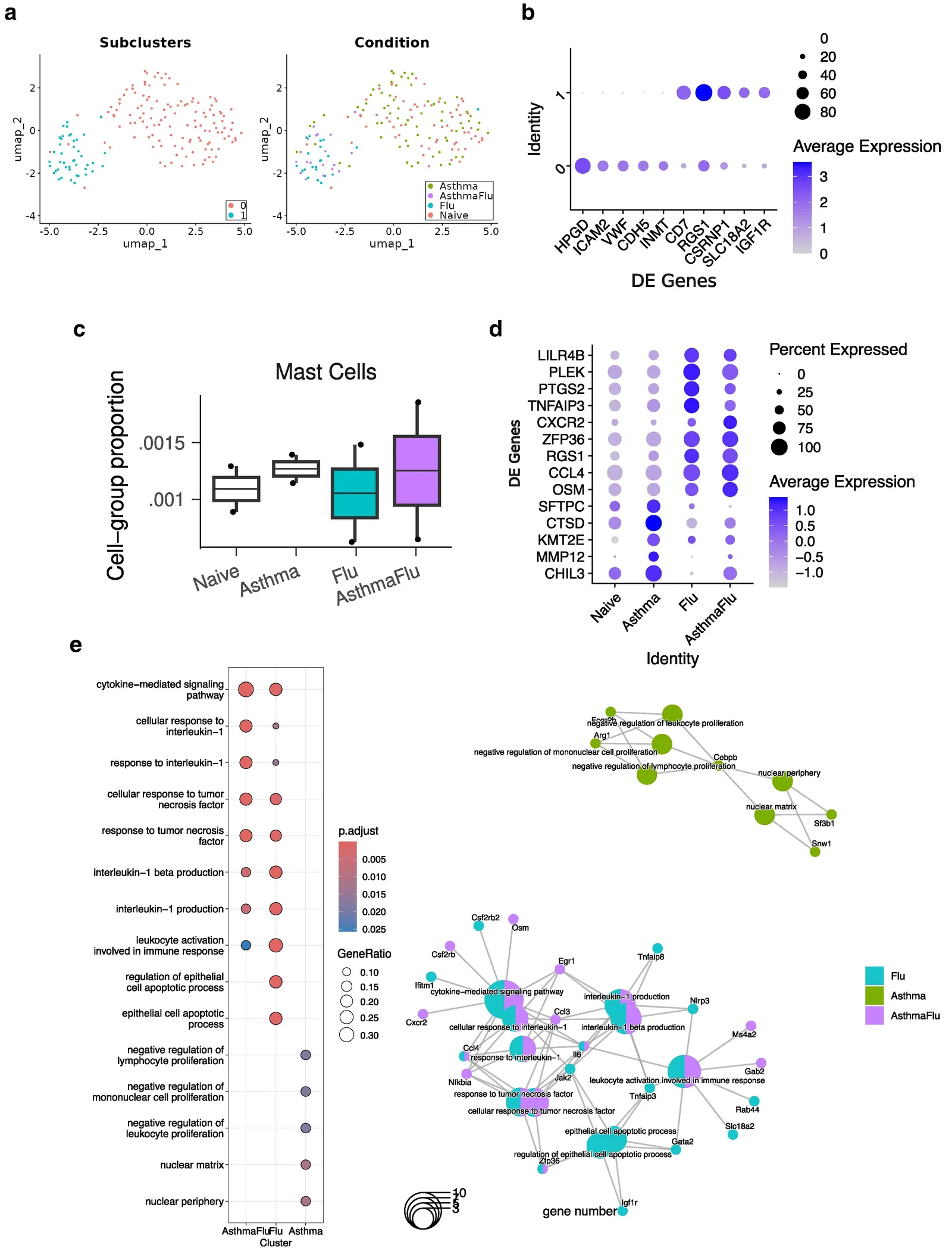
Transcriptomic and compositional analysis of MCs across conditions. (a) UMAP reanalysis and reclustering (left) of mast cells after subsetting. Conditions are represented as a color for each MC (right). (b) Top marker genes for each cluster represented as a dot plot. (c) Compositional analysis of proportions of MCs relative to all cells for each sample. (d) Expression of top genes upregulated in each of asthma, flu, and asthma + flu comparisons vs naïve, calculated by MAST statistical analysis. (e) Comparisons of top pathways associated with each condition vs. naïve comparison.

Despite the striking separation by condition observed in the clustering analysis, we did not observe strong condition-specific changes in MC abundance in contrast to EOS (Fig. 4c), suggesting that MC numbers are stable across disease conditions but have differing transcriptomes.

To more robustly assess gene expression changes in each disease condition relative to naïve, we again took a differential expression approach using MAST. In agreement with the clustering analysis, many of the top genes upregulated in the asthma + flu and flu conditions were shared between these two conditions, whereas the asthma vs naïve comparison produced distinct genes that were not generally upregulated in the virus-infected conditions (Fig. 4d). The genes shared between these infected conditions vs naïve comparisons produced strong associations with pathways related to IL-1 signaling and leukocyte activation, whereas the asthma vs naïve comparison did not exhibit strong upregulation of these inflammatory pathways (Fig. 4e). Interestingly, the flu vs naïve comparison had upregulation of some pathways related to epithelial cell apoptosis and inflammatory responses, whereas the asthma + flu condition did not, raising the tantalizing possibility that allergic conditioning in MCs may be beneficial to airway epithelial cells during IAV infection (Fig. 4e and Figure S3a). The asthma + flu condition also showed some pathways related to EOS, neutrophil, and general granulocyte chemotaxis, suggesting a greater role for cell-cell signaling in this condition (Figure S3b). The asthma alone vs naïve comparison exhibited a quite distinct gene signature and included the top genes *Mmp12* and *Chil3* that were also upregulated in the asthma condition among EOS and in the pseudotime gene trend 2 (Figs. 2f, 3d, and 4d). These similarities may indicate that EOS and MCs respond similarly during asthma.

Among the most upregulated pathways in the asthma vs naïve comparison, there was an interconnected network of pathways related to the negative regulation of lymphocyte proliferation, as well as some pathways indicating components of the nuclear matrix/periphery (Fig. 4e, Figure S3c). The similarities and differences between the activated pathways and proliferation of MCs for each disease state indicates tight regulation of the role of MCs in responding to these diseases.

### 3.6. An Alox15^+^ macrophage population has a role in signaling to EOS and MCs in asthma, yet is depleted upon infection

We next turned our attention to the Alox15^+^ macrophage population that composed the last of the three subclusters. Although this population was distinguished from other cells by high *Alox15* expression, similar to the EOS cluster, we asked which additional transcriptional signatures differentiated them from the other, more abundant macrophage populations (Fig. 1c). We performed cell-level Wilcoxon RST differential expression analysis of this population vs all other annotated macrophages in the dataset (Fig. 5a). As noted earlier, *Prg4* and *Cd5l* are major markers present on this population that are absent on other macrophages; we also observed high expression of *Vsig4*, *Cxcl13*, and *Timd4*. Conversely, top marker genes present to some extent on other macrophage populations, such as *Ms4a7*, *Fabp1*, *Cidec*, *Clec1b*, and *Pparg*, were generally absent from the *Alox15*^+^ population. To elucidate biological functions of the genes upregulated in this population, we ran pathway analysis, which indicated an upregulation of pathways related to humoral immune response, complement activation, and organ induction (Fig. 5b), suggesting there are some distinct disease-relevant functions of the *Alox15*^+^ population in contrast to the general macrophage populations of the lung.

**Figure 5:**
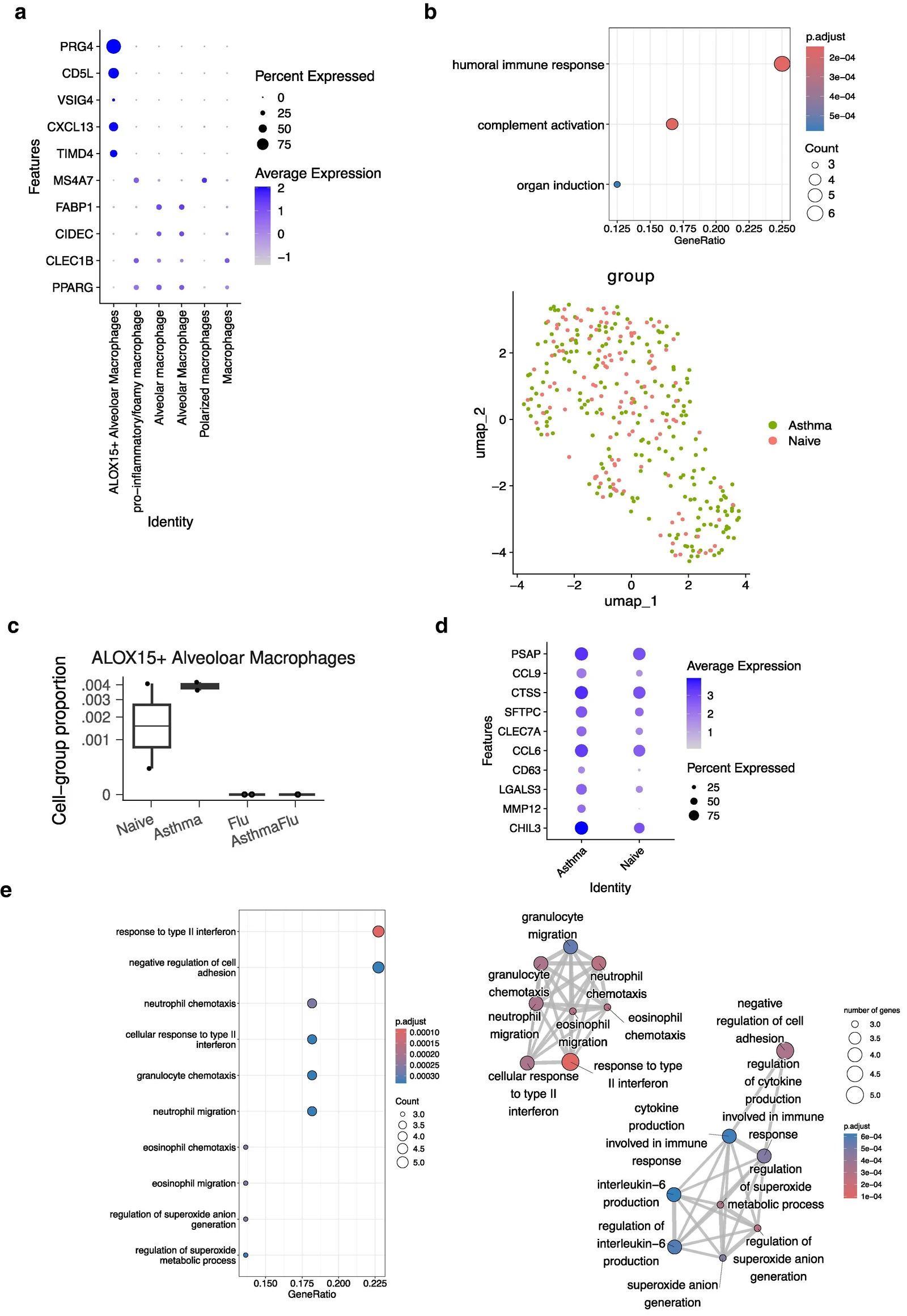
Transcriptomic and compositional analysis of *Alox15*^+^ macrophages across conditions. (a) Top marker genes of *Alox15*^+^ macrophages vs other macrophage types in the dataset. (b) Top pathways associated with marker genes of the *Alox15*^+^ macrophages. (c) UMAP reanalysis and of *Alox15^+^* macrophages after subsetting, with condition represented as color for each cell. (d) Compositional analysis of proportions of *Alox15*^+^ macrophages relative to all cells for each sample. (e) Top genes by MAST analysis for asthma vs naïve comparisons. No genes met the threshold for statistical significance at an adjusted *P* value (p.adjust) <0.05, so thresholds were relaxed to an unadjusted *P* value <0.05. (f) Comparisons of top pathways associated with asthma vs naïve differential expression at unadjusted *P* < 0.05.

We attempted to subcluster these cells (Fig. 5c); however, although the Wilcoxon RST identified several significant marker genes, these genes were generally expressed in only a small fraction of cells and did not define biologically meaningful differences among the resulting cell populations. More striking differences were observed when comparing the abundance of these cells across conditions where cells from the flu and asthma + flu conditions were absent from this population, indicating a depletion of *Alox15^+^* macrophages in flu-infected conditions or a small presence below the detection limit for these conditions (Fig. 5d).

We focused exclusively, therefore, on the asthma vs naïve comparison for our MAST analysis. In contrast to the MC populations, we observed only weak differences between asthma and naïve conditions by differential expression (despite a larger number of available cells for comparison), with no genes rising to the level of statistical significance at a false discovery rate < 0.05 (Fig. 5e). Nevertheless, by relaxing our threshold for inclusion to use the unadjusted *P* value of < 0.05, we took the top 25 genes and ran pathway analysis as before. Here, we observed pathways related to type II interferon (IFN) response and negative regulation of cell adhesion as among the most upregulated pathways (Fig. 5f, left). Additionally, there was a cluster of pathways related to superoxide metabolism and chemokine production involved in immune responses, and an additional cluster was composed primarily of pathways related to migration and chemotaxis (Fig. 5f, right). This indicates that these cells are likely to form interaction networks to regulate movement of other cell types, including granulocytes like EOS and MCs in asthma while also being directly involved in immune response and regulation.

### 3.7. Interactions between cell types depend on disease state

To identify potential ligand-receptor communication among these cell populations, we ran CellChat to assess ligand-receptor pairs that may be co-expressed in these cell types. Much of the signaling centered on the *Alox15*^+^ alveolar macrophages, with these being the greatest signal senders and receivers in the naïve and asthma conditions (Fig. 6a). These signals were largely targeted at communication within these macrophages, through pathways like Thbs1-Cd36, App-Cd74, and Fn1-Itgam/Itgb2 (Fig. 6b). In the naïve condition, Thbs1-Cd36 was the largest contributor to signaling from these macrophages. However, in the asthma condition, App and Fn1 axes become more prominent as senders and broadened their signaling to include all three cell types as receivers, indicating that this macrophage population may have a role in regulating both MC and EOS activities in asthma. The signaling activities of EOS and MCs in these conditions also change subtly, with a slight diversification of the signaling in the asthma condition (Fig. 6b). EOS both sent and received more signals than MCs in both the naïve and asthma conditions (Fig. 6a).

**Figure 6:**
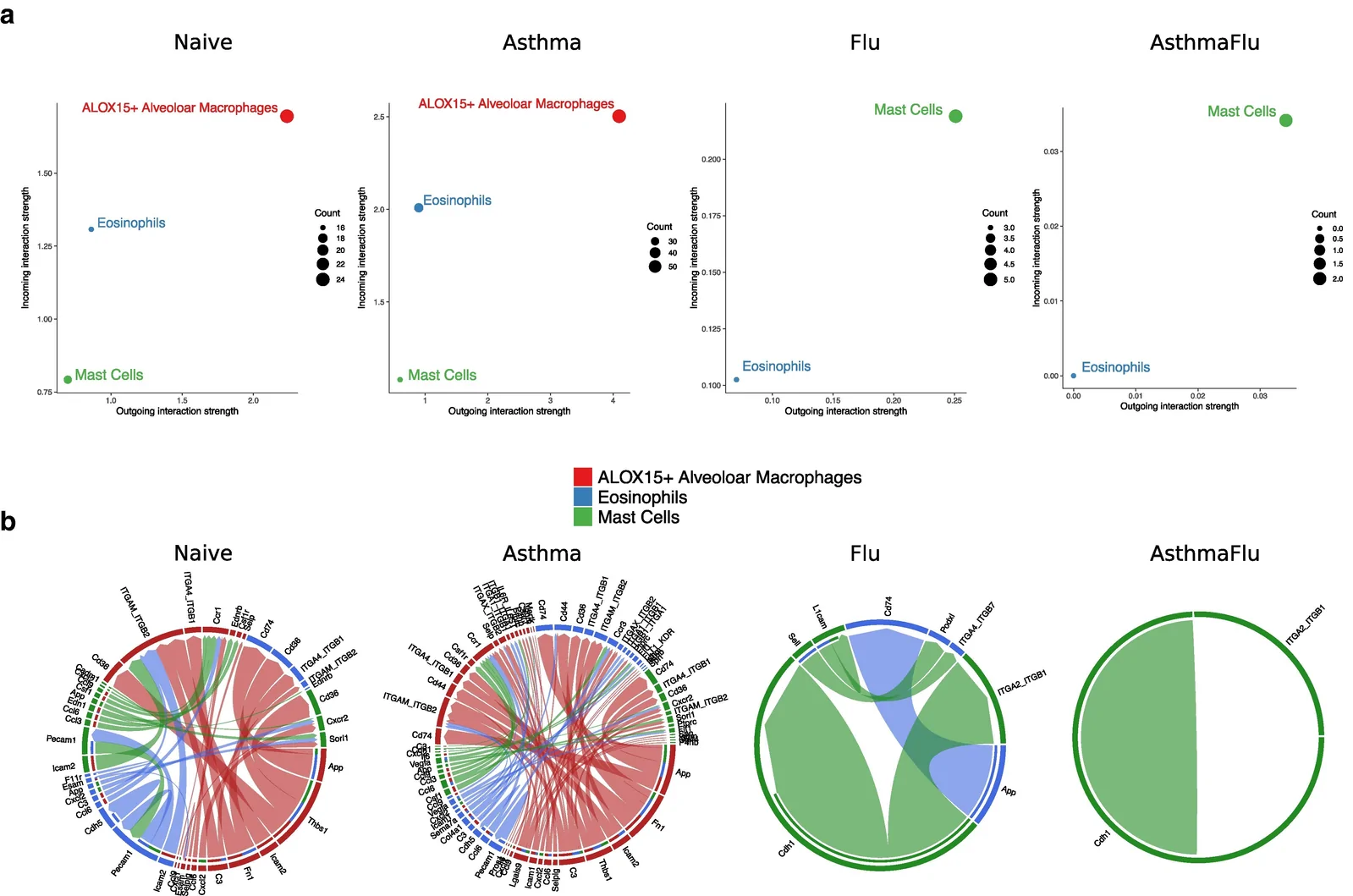
Cell-cell communication analysis of scRNA-seq. (a) Dot plots showing the outgoing and incoming signal strength from CellChat analysis of scRNA-seq data, plotting MCs, Alox15^+^ macrophages, and EOS. Interaction data are split by disease condition. (b) Circle diagrams show signaling ligand-receptor pairs active among the three cell populations of interest. Perimeter line width is proportional to signal strength.

Interestingly, with the *Alox15*^+^ macrophages absent in the flu and asthma + flu conditions, signal sending and receiving between EOS and MCs dropped precipitously, with no outgoing signaling from EOS to MCs detected, and only L1cam-Itga4/Itgb7 and Sell-Podxl signals being received by EOS. In the asthma + flu condition, no signaling by EOS to MCs or to themselves was detected, and only Cdh1 signaling was present by MCs to themselves (Fig. 6b). These drastic changes in flu-infected conditions point to disruptions in the network of signaling interactions among these three cell types and the important role of their coordination in healthy tissues.

### 3.8. ALOX15 is enriched in EOS but also is abundant in non-EOS

Given the evidence of these interactions between *Alox15* expressing macrophages and EOS in disease, and the expression of *Alox15* on some EOS in our dataset (Fig. 1e), we questioned whether *Alox15* expression itself was an indicator of a particular spatial niche within diseased tissue. Therefore, we performed immunofluorescence staining to visualize the locations of ALOX15 and mEARs (Fig. 7a). This experiment was performed across all four disease conditions, with three tissue sections (biological replicates) per slide and between 29 and 125 40 × FOVs per section for a total of 1,051 images (Figure S4a). In addition to our proteins of interest, we additionally captured DAPI fluorescence to stain nuclei and enable nuclear segmentation via StarDist.^26^ After segmentation and boundary expansion was performed on all images, we analyzed the resulting 815,581 cells to quantify protein abundance in our samples. We then used thresholding to assign positive cells based on abundance of mEARs and ALOX15 (Figure S4b).

**Figure 7:**
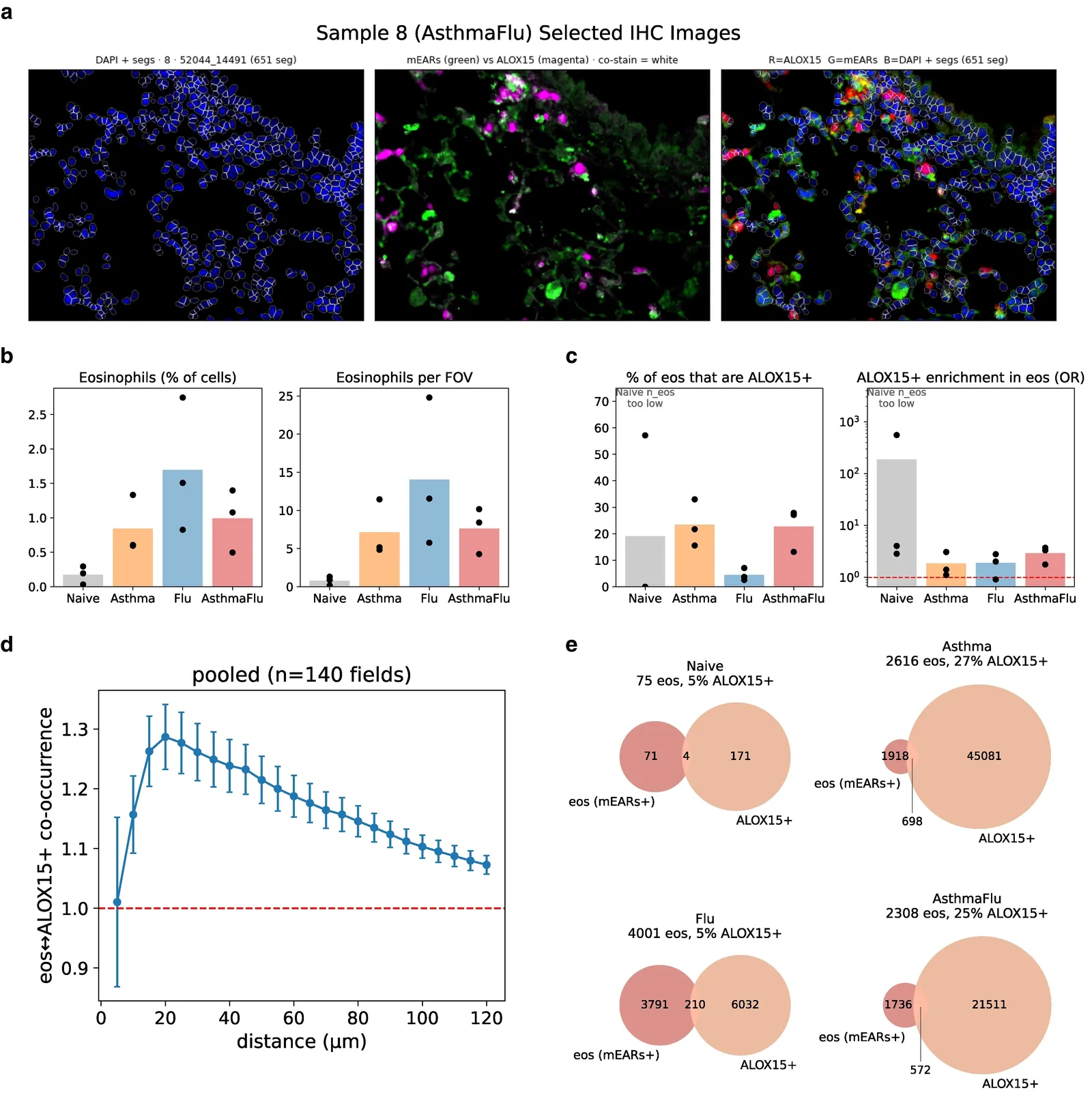
Immunofluorescence of EOS and ALOX15 expression in each disease state. (a) Selected images depicting immunofluorescence stains and cell segmentation in one FOV from sample 8 (asthma + flu). DAPI stain in blue and StarDist cell segmentations in white (left). mEars (green) and ALOX15 (magenta) stains from the same section (middle). A merge showing ALOX15, mEARs, DAPI, and cell segmentations (right). (b) The percentage of mEARS + EOS of all cells identified across FOVs in each sample (left). The average number of mEARs + EOS identified per FOV in each disease condition (right). (c) Percentages of identified EOS that are ALOX15^+^ (left). Note that low numbers of EOS in naïve samples introduce noise. ALOX15^+^ enrichment in EOS plotting the odds ratio (OR) of ALOX15-positivity in EOS vs non-EOS (mEARs-negative) cells (right). (d) Squidpy-calculated average co-occurrence of EOS and ALOX15^+^ cells across spatial distances. Positive co-occurrence indicates grouping or clustering of cells near one another. (e) Venn diagrams showing overlap between mEAR-positive EOS and ALOX15^+^ cells, indicating a large population of ALOX15^+^ cells and a portion of EOS expressing ALOX15.

Although FOVs were chosen intentionally to capture areas where EOS and ALOX15 abundances were high (and this selection bias does not enable global conclusions), we noticed that the disease condition FOVs contained greater percentages of EOS than the naïve condition (Fig. 7b, left), with average EOS counts per FOV ranging from 0.784% in naïve to 14.035% in flu, whereas asthma and asthma + flu had average counts at 7.151% and 7.619%, respectively (Fig. 7b, right). When we further assessed the percentages of mEAR-positive EOS that were additionally ALOX15-positive, we noticed that the asthma and asthma + flu conditions had average ALOX15-positivity percentages of 23.416%, and 22.71%, respectively, whereas flu had a much lower positivity rate of 4.429%. Although the naïve group had an average ALOX15-positivity rate near 20, the numbers from this sample were affected by the low absolute counts of EOS (only 4 ALOX15^+^mEAR^+^ cells were detected from a single replicate), which amplified noise (Fig. 7c, left).

We additionally tested enrichment of ALOX15^+^ in EOS by calculating the odds ratio of ALOX15 positivity in EOS vs non-EOS and noted it was positive across all disease conditions, indicating an enrichment (Fig. 7c, right). Furthermore, we assessed spatial co-occurrence of ALOX15^+^ cells and EOS across all FOVs, regardless of condition, which demonstrated enrichment > 1 across increasing distance ranges (Fig. 7d) and can be interpreted as clustering of ALOX15^+^ cells and EOS spatially across FOVs.

Strikingly, however, in addition to ALOX15^+^ EOS, there was a greater abundance of mEAR-negative cells that also expressed ALOX15, with asthma and asthma + flu samples having an order of magnitude more of other ALOX15^+^ cells than EOS (Fig. 7e). Although the identities of these cells could not be determined at scale from this experiment, we tested whether some of these non-EOS ALOX15^+^ cells may correspond to the *Alox15* expressing macrophages observed in scRNA-seq.

### 3.9. Spatial transcriptomics identifies an Alox15^+^ niche in disease states

We performed 10x Genomics Xenium in situ spatial transcriptomics using a panel of ∼ 5,000 mouse genes. We performed this experiment on consecutive sections from the same FFPE tissue blocks as the immunohistochemical assay, and we used the same experimental design of three tissue slices (biological replicates) per disease condition, assayed across four Xenium slides. This design allowed direct visualization of tissues and comparison with scRNA-seq, including label transfer of identified cell types (Figure S5a). We performed similar data processing as our scRNA-seq analysis, including normalization, cell quality filtering, and Harmony integration across samples. Because Xenium performs cell segmentation on microscope images, but not every cell has robust transcript detection, we filtered out low-quality cells with fewer than 28 unique molecular identifiers detected, to enable more effective UMAP generation and label transfer from our scRNA-seq dataset. Every sample remained represented; however, varying quality between samples caused some to contribute more high-quality cells than others (Figure S5c).

We performed the label transfer by using a CellTypist model trained on our scRNA-seq dataset and predicting cell identity on an individual cell basis in the Xenium data, using only the genes shared between assays (Fig. 8a and Figure S5a). As expected, many similar marker genes were detected for the predicted populations (Figure S5b). Using this method, we identified putative EOS across conditions, with disease states showing elevated EOS proportions compared with naïve samples (Fig. 8b). Although CellTypist label transfer predictions should not be regarded as definitive cell identities, we note that the predicted EOS were largely clustered together in the UMAP (Figure S5d, left), indicating broad transcriptional similarity, with some other populations spread throughout, possibly related to EOS presence in different spatial niches. To assess label transfer, we also manually annotated selected regions of each sample to identify ∼ 6,000 EOS based on aligned hematoxylin and eosin–stained images. Although manual annotation identified many EOS that were not detected by automated label transfer and vice versa (Figure S5e), the distribution of identified EOS across the UMAP was broadly consistent (Figure S5d, right), with areas of increased density corresponding to similar regions. Many EOS (*n* ∼ 3,500) within the manually annotated regions fell below the 28 unique molecular identifier cutoff used for label transfer, indicating a sensitivity limitation of the Xenium assay, and highlighting that transcriptomic detection of EOS is limited to a subset of true EOS in the tissue.

**Figure 8:**
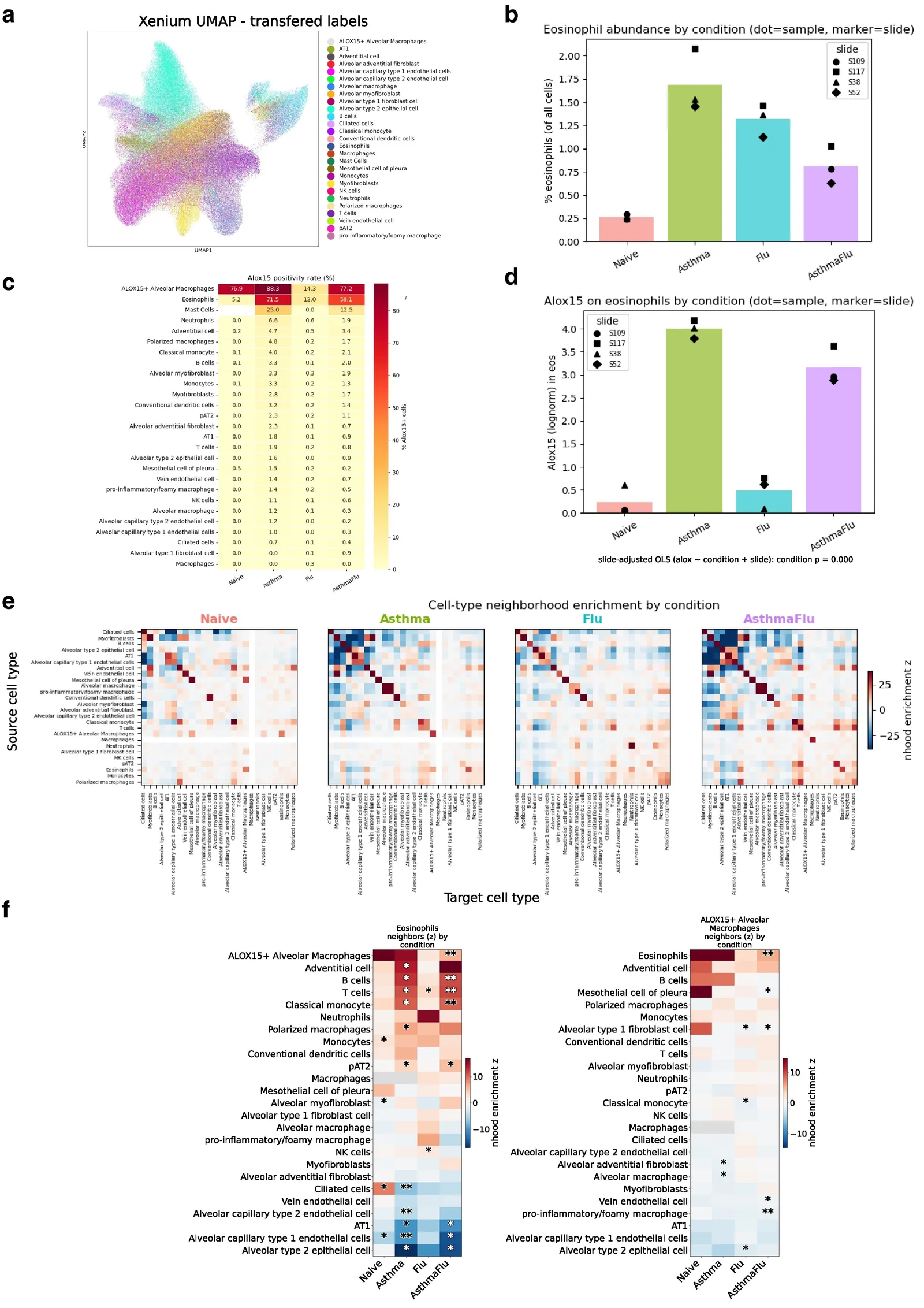
In situ spatial transcriptomics on lung sections to measure *Alox15* positivity and detect spatial niches. (a) UMAP plot calculated on gene expression from cells segmented by Xenium spatial transcriptomics. Samples were quality filtered and integrated into a single UMAP, and cell-type labels were transferred via CellTypist using a model trained on this study's scRNA-seq data. (b) Predicted EOS abundance by condition. Different Xenium slides (indicated by the last two digits of a serial number) were plotted and averaged to calculate average EOS percentage across conditions. (c) *Alox15*-positivity rate among different predicted cell types from spatial transcriptomics. Percentages are presented as a total of all cells in that condition. (d) *Alox15* gene expression in predicted EOS measured by spatial transcriptomics. Significance of condition as a factor was calculated using an ordinary least squares (OLS) model correcting for slide origin and tested using analysis of variance. (e) Heat maps of Squidpy calculated average neighborhood (nhood) enrichment *z* score, with source cell type indicated in the rows and target cell type in the columns. A target cell type is considered enriched in the surrounding environment of a source cell if the *z* score is positive and de-enriched if the *z* score is negative. Neighborhood enrichment *z* scores were calculated independently for each sample and averaged per condition. (f) Average neighborhood enrichment score with EOS as the source cell type (left). Average neighborhood enrichment score with *Alox15*^+^ macrophages as the source cell type (right). Significance was tested against the null hypothesis that the mean *z* score equals 0, using a one-sample *t* test. In heat map cells, *P < 0.05, **P < 0.01, ***P < 0.001. NK, natural killer.

Label transfer also predicted a limited number of *Alox15*^+^ macrophages (Figure S5d, bottom) across all three disease conditions (in contrast to scRNA-seq, where detection was limited to the asthma condition). These cells expressed high levels of *Alox15*, similar to EOS; however, they had distinct upregulated genes, such as *C4b*, *Fn1*, and *Grn*, which are involved in innate immunity and extracellular matrix remodeling (Figure S5b). A very small number of MCs were also predicted; however, their detection rates were too low for further analysis.

Next, we measured the *Alox15* positivity rate as a percentage of total cells identified in each condition, and we noticed that, although *Alox15*^+^ macrophages had the highest positivity rates overall, EOS also had high percentages of *Alox15* positivity (Fig. 8c), and indeed, both the percentage-positive and relative amounts of expression (Fig. 8d) recapitulated the pattern observed by immunofluorescence, in which asthma and asthma + flu samples had elevated EOS *Alox15*, and flu samples had lower levels in comparison. Controlling for slide identity, disease condition was a statistically significant factor in predicting *Alox15* expression in EOS.

Based on these data, we hypothesize that *Alox15* plays a role both in macrophages and in EOS response to asthma-induced inflammation. Although our data provide some evidence for this hypothesis, additional, more sensitive experiments are needed to fully identify EOS distributions and *Alox15* expression patterns across lung tissue in these disease conditions. It will also be important to place changes in these cell populations in the context of changes between disease conditions in all cell types (Figure S5f), an analysis that was beyond the scope of this work.

### 3.10. Cell distributions identify disease-specific spatial niches

We next examined the spatial distribution of predicted cell types across the tissues to determine whether neighborhood enrichment analysis could identify patterns of spatial organization and cell type colocalization. We calculated neighborhood enrichment *z* score using Squidpy and ran this analysis separately for each tissue sample (filtering out tissue sections with fewer than 10 cells of a given type detected, and removing cell populations for which only one tissue section met this cutoff). Then we aggregated results by averaging on the condition level (Fig. 8e). This score provides a relative measure of how abundant other cell types are within a local neighborhood vs a set of randomly permuted background cells. A higher score indicates enrichment of a target cell type in the vicinity of the source cell type, controlling for relative abundance. Naïve samples had lower levels of spatial organization by cell type, with alveolar capillary type 1 endothelial cells and alveolar type I (AT1) cells showing colocalization, as well as some other individual groups of cells such as myofibroblasts, vein endothelial cells, and adventitial cells. Disease samples exhibited some reorganization of the tissue, with cells such as EOS, monocytes, and polarized macrophages increasing in enrichment score, in addition to the emergence of other pairs like monocytes and T cells, and alveolar and pro-inflammatory/foamy macrophages, which became much more prominent in asthma and asthma + flu conditions and were moderately increased in the flu-only condition (Fig. 8e). Interestingly, *Alox15*^+^ macrophages and EOS had positive *z* scores across all tissue types.

To more specifically investigate colocalization of *Alox15*^+^ macrophages and EOS, we analyzed their specific neighbors across conditions and ran a univariate *t* test to evaluate whether neighborhood enrichment was significantly different than 0. Although the very low numbers of identified *Alox15*^+^ macrophages limited our ability to detect statistically significant differences, there was detectable neighbor enrichment between EOS and *Alox15*^+^ macrophages in the asthma + flu condition (Fig. 8f), demonstrating preferential association between these cell types in a disease condition. Interestingly, EOS also appeared to show differential localization in asthma and asthma + flu conditions, with significant neighbor enrichment for B cells, T cells, and classical monocytes, and de-enrichment for AT1, alveolar capillary type 1 endothelial cells, and alveolar type 2 epithelial cells in these conditions (Fig. 8f, left). This may be related to the inflammatory response in asthma. In the flu condition, the only significant enrichment in neighbors was for T cells and natural killer cells, which may correspond to an antiviral immune response to infection. Interestingly, natural killer cells did not show this same enrichment in the asthma + flu condition. Although the low numbers make *Alox15*^+^ macrophage neighbors more difficult to interpret, there was significant de-enrichment for other macrophage types in the asthma groups (Fig. 8f, right), possibly indicating that these cells have a distinct localization from other macrophages in asthma. Additional, higher-powered studies are needed, however, to fully clarify the spatial niche and localization of this distinct macrophage population.

### 3.11. Spatial cell-cell communication analysis refines communication pathways between EOS and Alox15^+^ macrophages

Given the significant colocalization of *Alox15*^+^ macrophages and EOS in at least one disease condition, we next used spatially informed cell-cell communication analysis to compare the major ligand-receptor pathways identified by Xenium with those detected by scRNA-seq. We used LIANA+^50^ software to score interactions with a bandwidth of 250 µm based on the mouse_consensus set of multiple ligand-receptor databases and focused first on interactions involving EOS as either senders or receivers. We scored the top pathways by average magnitude_rank across all samples to get a global view of EOS signaling. As senders, EOS had a limited number of strong interactions, mostly centered around the App pathways, signaling to Lrp1 or Ager receptors on several cell types, including alveolar macrophages, myofibroblasts, and adventitial cells (Fig. 9a). The average magnitude_rank of App→Lrp1 signaling to *Alox15*^+^ macrophages was among the top signaling/interaction, but much lower in lr_mean signal than other cell types, consistent with limited signaling/interaction from EOS to these macrophages compared with other cell types. We also aggregated signaling pathways by disease condition, and although there was some variation between conditions, such as in the asthma + flu condition, strong differences were not observed (Figure S6a, left).

**Figure 9:**
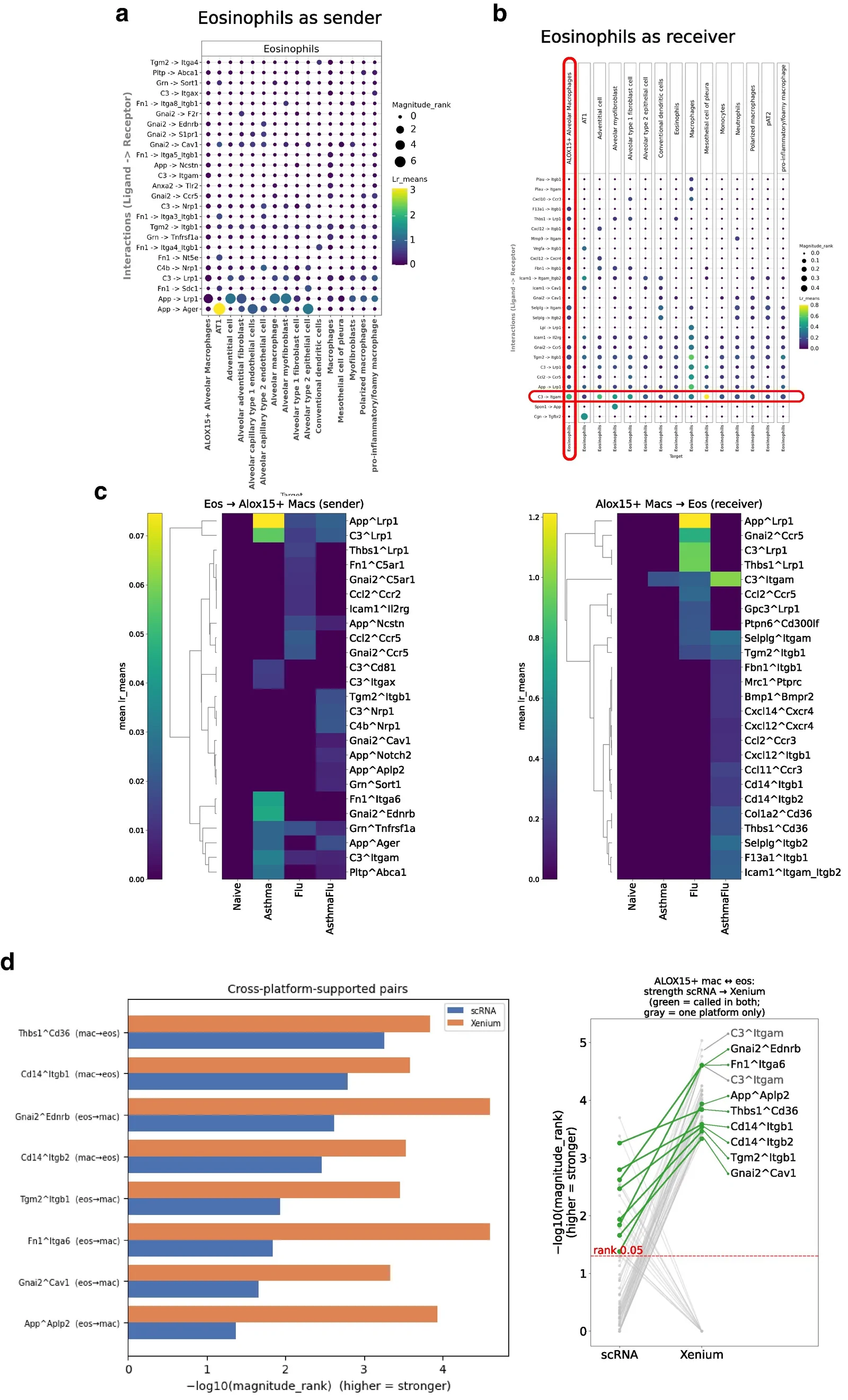
LIANA+ spatial cell-cell communication analysis for spatial transcriptomics and comparing to scRNA-seq. (a) A dot plot showing LIANA+ top ligand-receptor interactions by magnitude_rank score, where EOS are the signal receiver. Dot size is proportional to -log_10_(magnitude_rank), with small magnitude rank indicating stronger interaction. Dot color is related to lr_mean (average ligand and receptor expression across cells). Top interactions were ranked by computing LIANA scores across each sample individually and averaging the scores for all samples. (b) Dot plots showing the top interactions with EOS as receivers. Highlighted is the *Alox15*^+^ macrophage C3→Itgam pathway, one of the top hits in all EOS. (c) Heat map showing average lr_mean for top pathways (left) with EOS as senders and *Alox15*^+^ macrophages (Macs) as receivers. Note the low overall expression with highest average lr_mean at 0.07. Heat map per disease condition with *Alox15*^+^ macrophages as senders and EOS as receivers. (d) Top cross-platform pathways identified as high ranking in both scRNA-seq LIANA analysis and spatial transcriptomic LIANA analysis. A bar chart comparing −log_10_(magnitude_rank) for each platform (left). A line plot showing which pathways signal was increased using spatial transcriptomic information and which pathways signal decreased (right).

When analyzing EOS as receivers of signals, however, *Alox15*^+^ macrophages were a top sender of complement C3→Itgam (Cd11b) signals (Fig. 9a, right). An example of colocalization of EOS and *Alox15*^+^ macrophages expressing these ligands and receptors is shown in Figure S5a. Other cell types also signaled to EOS through this pathway at similar strength, including adventitial cells, myofibroblasts, mesothelial cells of pleura, and AT1 fibroblasts—cells that are known to be involved in the structural basis of inflammation in the disease conditions used in our study. When we broke these interactions down by disease condition, we observed that the C3→Itgam interaction from *Alox15*^+^ macrophages predominated in the asthma + flu condition (Figure S6a, right), the same condition in which we found significant colocalization of *Alox15*^+^ macrophages and EOS. In addition, mesothelial cells of pleura signal strongly through this pathway in asthma + flu, as well as adventitial cells, and some fibroblast and myofibroblast populations, indicating general complement and innate immune signaling in the spatial niche occupied by these EOS. The flu condition also had some uniquely upregulated pathways, including C3→Lrp1, App→Lrp1, Vwf→Lrp1, and Icam1→Il2rg from mesothelial cells of pleura, *Alox15*^+^ macrophages, general macrophages, and others (Figure S6a, right). This is notable because while some of the same ligands are expressed on sender cells in the flu condition, EOS appear to have upregulation of other receptors to receive these signals, suggesting the possibility that EOS may use different signaling programs in the flu and asthma + flu conditions.

*Alox15* ^+^ macrophages also signal to a broad range of cell types across conditions, although the leading pathways generally showed less-pronounced disease condition–specific differences (Figure S6b). As receivers of signals, nearly all the top pathways for *Alox15*^+^ macrophages came from *Alox15*^+^ macrophages themselves, with the naïve condition having upregulation of Fn1 and C4b pathways. These results, however, must be interpreted with caution due to the low numbers of *Alox15*^+^ macrophages detected in the naïve condition (Figure S6d).

We directly compared top signaling pathways involving *Alox15*^+^ macrophages and EOS in both directions (Fig. 9c). As noted earlier, only weak signaling was detected from EOS to *Alox15*^+^ macrophages in any condition, with a maximum average lr_means score of 0.07 (Fig. 9c). In the other direction, with EOS as receivers, flu showed distinct pathways upregulated, many with Lrp1 as a receptor, consistent with the signaling with other cell types. C3→Itgam was the pathway with the highest average lr_mean score for the asthma + flu condition. Together, these results support coordinated signaling between *Alox15*^+^ macrophages and EOS, with the specific ligand-receptor pairs varying by disease condition.

Finally, we compared top *Alox15*^+^ macrophage/EOS ligand-receptor pairs identified in scRNA-seq with those detected in spatial transcriptomics. To ensure a fair comparison, we reran scRNA-seq cell-cell communication analysis using LIANA and the same mouse_consensus database used for spatial transcriptomics. Many of the same top pathways were shared between the two platforms, though each had distinct detections not found in the other (Figure S6c). We used a magnitude_rank cutoff of 0.05 to define top ligand-receptor pairs in each platform and found a set of eight interactions that were high across both platforms, lending increased confidence in our results (Fig. 9d). The C3→Itgam pathway just barely did not meet this 0.05 cutoff for scRNA-seq, but we note that its signal may be diluted by being a disease-enriched interaction for the asthma + flu condition, for which we did not detect any *Alox15*^+^ macrophages in scRNA-seq. However, when looking at C3 and Itgam expression from scRNA-seq (Figure S6, bottom), we note that many of the same cell types involved in the signaling detected by spatial transcriptomics express these genes. The eight highest-ranking interactions shared between the spatial transcriptomics and scRNA-seq analyses identify candidate molecular pathways that may coordinate communication between *Alox15*^+^ macrophages and EOS across disease conditions. More studies are needed to validate these interactions and elucidate their roles in shaping EOS responses in the lung during health and disease.

## 4. Discussion

EOS heterogeneity in tissues is no longer speculative. Tissue-resident and inflammatory EOS subsets were described in 2016 by Mesnil et al.,^6^ and recent single-cell atlases have confirmed transcriptional diversification in EOS across tissues and diseases.^11–14^ What has remained unclear is how allergic inflammation shapes EOS states during respiratory viral infection and whether canonical antiviral programs are preserved, amplified, or redirected in this setting. Over the past decade, we established that EOS respond directly to IAV, become activated and undergo piecemeal degranulation,^17^ alter their phenotype and transcriptome,^7^ protect airway epithelial cells,^16^ migrate to draining lymph nodes, and present viral antigen to promote CD8^+^ T-cell priming, clonal expansion, and epigenetic changes.^7,16^ Here, we extend those observations by defining disease-associated transcriptional states in EOS and placing these cells within the broader cellular and spatial contexts of the asthma and influenza co-morbid lung.

Our data demonstrate that allergic conditioning substantially alters the EOS transcriptional response to IAV infection. A clear difference in EOS transcriptional programs was noted between disease conditions. EOS in the flu-only condition preferentially expressed canonical IFN-stimulated gene programs (eg *Isg15*, *Gbp2*, *Samhd1*), consistent with our prior evidence that EOS directly respond to IAV.^7^ In contrast, EOS in the asthma + flu condition were enriched for programs associated with migration/chemotaxis, adhesion, inflammatory regulation, and metabolic remodeling, with weaker induction of the canonical antiviral programs. For example, *Il1rn*, which codes for IL-1 receptor antagonist (IL-1Ra), was abundantly expressed in asthma + flu EOS, suggesting the induction of an anti-inflammatory program that may restrain IL-1–driven inflammatory responses during influenza. EOS-derived IL-1Ra suppresses Th17 differentiation in the intestine,^51^ supporting a broader immunoregulatory role for this pathway. These findings do not indicate a complete loss of antiviral capacity in the co-morbid condition but rather suggest that the allergic lung microenvironment changes the balance of EOS transcriptional programs during infection. This interpretation is consistent with the local immunity and/or remodeling/repair (LIAR) hypothesis.^52^ Perhaps a transition from antiviral to remodeling or repair states are more hierarchical than additive, suggesting distinct functional roles for the subclusters of EOS identified here.

The enrichment of the human asthma-associated EosHigh signature^14^ within our asthma + flu EOS provides a useful translational link between our murine model and human EOS clusters identified in human lung disease.^13,14^ The EosHigh signature was greatest in the EOS state enriched in asthma + flu condition, whereas the EosLow signature remained comparatively weak across conditions. These findings suggest that allergic inflammation establishes a conserved EOS transcriptional state that persists during viral infection. However, the functional significance of this signature during IAV infection remains unresolved because the present work does not establish whether the EosHigh program promotes host defense, contributes to inflammation, or supports tissue repair. These questions form the foundation of our ongoing work.

Pseudotime analysis further supported the presence of a transcriptional continuum among lung EOS. EOS from naïve and asthma conditions were distributed toward early regions of the inferred trajectory, whereas subsets of the asthma + flu EOS occupied more transcriptionally divergent positions. Early trajectory changes included activation of antigen-presentation pathways, consistent with our prior work.^17^ However, these pathways declined at later pseudotime states, whereas chemotactic and metabolic programs intensified. Pathways associated with viral life-cycle regulation and cytotoxic activity decreased along the trajectory. This pattern is more consistent with functional diversion or regulatory remodeling than with sustained antiviral activation. Similar exhaustion-like transcriptional states have been described in other innate leukocytes during chronic or dysregulated inflammatory responses,^53,54^ although EOS have not been viewed previously through this lens. We are currently investigating this hypothesis by purifying unaltered EOS from the lungs of mice in this model to define their functional properties and to determine whether allergic conditioning redirects antiviral eosinophil programs toward tissue-protective or immunoregulatory states. It is plausible that allergic conditioning pushes EOS toward programs that reduce tissue damage (and therefore reduce flu-mediated morbidity, which we have shown to occur in the asthma + flu group^18^), whereas viral infection is handled by other immune mechanisms such as IFN-λ at the epithelium.^18,55^ Overall, these data suggest that asthma conditioning reprograms the EOS antiviral strategy in response to IAV, decoupling classical IFN and IFN-stimulated gene transcription from pathways linked to trafficking, tissue interaction, and immune orchestration.

Spatial transcriptomic and imaging analyses provided orthogonal validation and tissue context for the single-cell findings. Xenium profiling confirmed condition-dependent differences in EOS abundance and transcriptional programs, and demonstrated that EOS *Alox15* expression was highest in conditions associated with T2 inflammation (asthma and asthma + flu). Arachidonate 15-lipoxygenase (LOX), a non-heme iron enzyme that oxygenates polyunsaturated fatty acids, is coded for by *Alox15*.^56^ EOS express *Alox15* and are a major source of 12/15-LOX–derived lipid mediators in mice.^44^ Quantitative imaging independently identified ALOX15^+^ EOS and showed nonrandom spatial juxtapositioning between EOS and other ALOX15^+^ cells in the lungs. Therefore, allergic conditioning in the lung appears to promote an ALOX15-associated EOS program that persists during IAV-induced inflammation. Importantly, these data show that the EOS states identified by scRNA-seq are embedded within disease-specific tissue environments rather than arising solely as artifacts of tissue dissociation or computational clustering.

The shared *Alox15* expression between an alveolar macrophage subset and EOS further raises the possibility that a lipid mediator–based crosstalk between EOS and macrophages occurs during co-morbidity. Disruption of this axis during IAV infection may contribute to the altered EOS states observed in asthma + flu. The importance of macrophages to lung health and their contribution to lung defense is vast.^57^ IAV counters innate immune defenses early during infection by targeting alveolar macrophages inducing apoptosis.^58^ Of the several lung macrophage populations we found in our model, the *Alox15*^+^ macrophages (enriched for complement activation and humoral immune pathways) were the most unique. ALOX15 has recently been implicated in antiviral lipid mediator pathways and resolution biology.^59^ Strikingly, *Alox15*^+^ macrophages were absent in the flu-only condition. Given their transcriptional enrichment for chemokine production and impact on IFN response pathways, these cells may participate in granulocyte orchestration or immune regulation in the allergic tissue. Their reduced recovery in the scRNA-seq dataset, may suggest the targeted disruption of a potentially protective or regulatory macrophage subset during infection.

Neighborhood enrichment and ligand-receptor analyses further suggested that EOS and *ALOX15*^+^ macrophages participate in a disease-dependent cellular niche. Their spatial association varied across conditions, and recurrent candidate interaction axes were supported in both the scRNA-seq and Xenium analyses. Although cross-platform identification of these pairs makes them stronger candidates for interactions, colocalization and coordinated ligand-receptor expression do not establish direct signaling, and these pathways require functional testing. Nonetheless, these cumulative data suggest that EOS behavior in the allergic lung is influenced by cell-intrinsic transcriptional programs that remain responsive to other stimuli (eg virus infection) and changes in neighboring cell populations.

Although MCs are difficult to study due to small numbers relative to other cells during lung inflammation, they are now known for their phenotypic and functional subsets.^60,61^ We have previously shown that they are abundant in the upper airway areas of the lungs in our mice.^62^ Here, we show that MCs are of unique transcriptional states in the model. IAV infection induced pathways for IL-1 signaling and leukocyte activation, consistent with a known role for MCs as amplifiers of inflammation during virus infections.^63,64^ Although MCs are classically viewed as dependent on immunoglobulin E (IgE) crosslinking to degranulate, the absence of detectable IgE in the lungs and serum of flu-only mice^22^ suggests that a broader immunomodulatory function of MCs, independent of IgE signaling, may be active in this setting.^65^ Pathways related to epithelial cell apoptosis were more prominent in MCs in the flu-only condition than MCs in the asthma + flu condition, raising the possibility that allergic conditioning modifies MC responses to infection. However, these pathway-level findings do not establish a direct effect of MCs on epithelial cell survival, which will require functional validation.

### 4.1. Study limitations and conclusion

Our prior work has clearly demonstrated marked temporal and kinetic changes in EOS phenotype and function in this model.^7,16,18^ However, this study captures a single snapshot of the EOS response during asthma and influenza co-morbidity. Therefore, longitudinal profiling at this depth will be required to determine whether transcriptional programs identified here are transient, sustained, or permanently reprogrammed in the allergic lung over the course of infection. The scRNA-seq analysis was also limited by the number of biological replicates per condition, and cell recovery in dissociation-based datasets may be influenced by viability and platform-specific capture efficiency, particularly in EOS. In addition, methodologies such as pseudotime and ligand-receptor analyses are inferential and do not prove chronological state transitions or direct cellular crosstalk. Cell isolation and functional validation of EOS states and *Alox15*^+^ macrophage population identified here will be required to define their causal contributions to allergic inflammation and antiviral host defense, barrier protection, and tissue remodeling. Integration of compositional modeling, mixed-effect differential expression, pathway network analysis, pseudotime inference, spatial transcriptomics, quantitative imaging, and cross-platform ligand-receptor analysis further demonstrated that EOS states occur within disease-specific cellular neighborhoods.

Collectively, our findings support a model in which allergic airway inflammation establishes a distinct transcriptional and spatial framework for EOS responses to IAV infection. These findings refine our understanding of EOS heterogeneity in asthma and viral infection and highlight the importance of both cell-intrinsic transcriptional state and tissue context in shaping host-defense outcomes in the lung mucosa.

## Supplementary Material

qiag120_Supplementary_Data

## Data Availability

Single-cell RNA-sequencing data and Xenium transcriptomic data have been made available as interactive data viewers using the University of California, Santa Cruz CellBrowser. These are available online at https://samarasinghe.medicine.wisc.edu/shared-data/. The data underlying this article will be shared on reasonable request to the corresponding author.
