# Supplementary material for: Single-cell and spatial transcriptomics define eosinophil heterogeneity during influenza A virus–induced exacerbation of fungal allergic asthma in C57BL/6 mice
Source: J Leukoc Biol. 2026 Sep 8;118(10):qiag120. doi: 10.1093/jleuko/qiag120 (PMC13655587; doi:10.1093/jleuko/qiag120)
Supplement: Supplementary file 1 [file qiag120_supplementary_data.pdf]

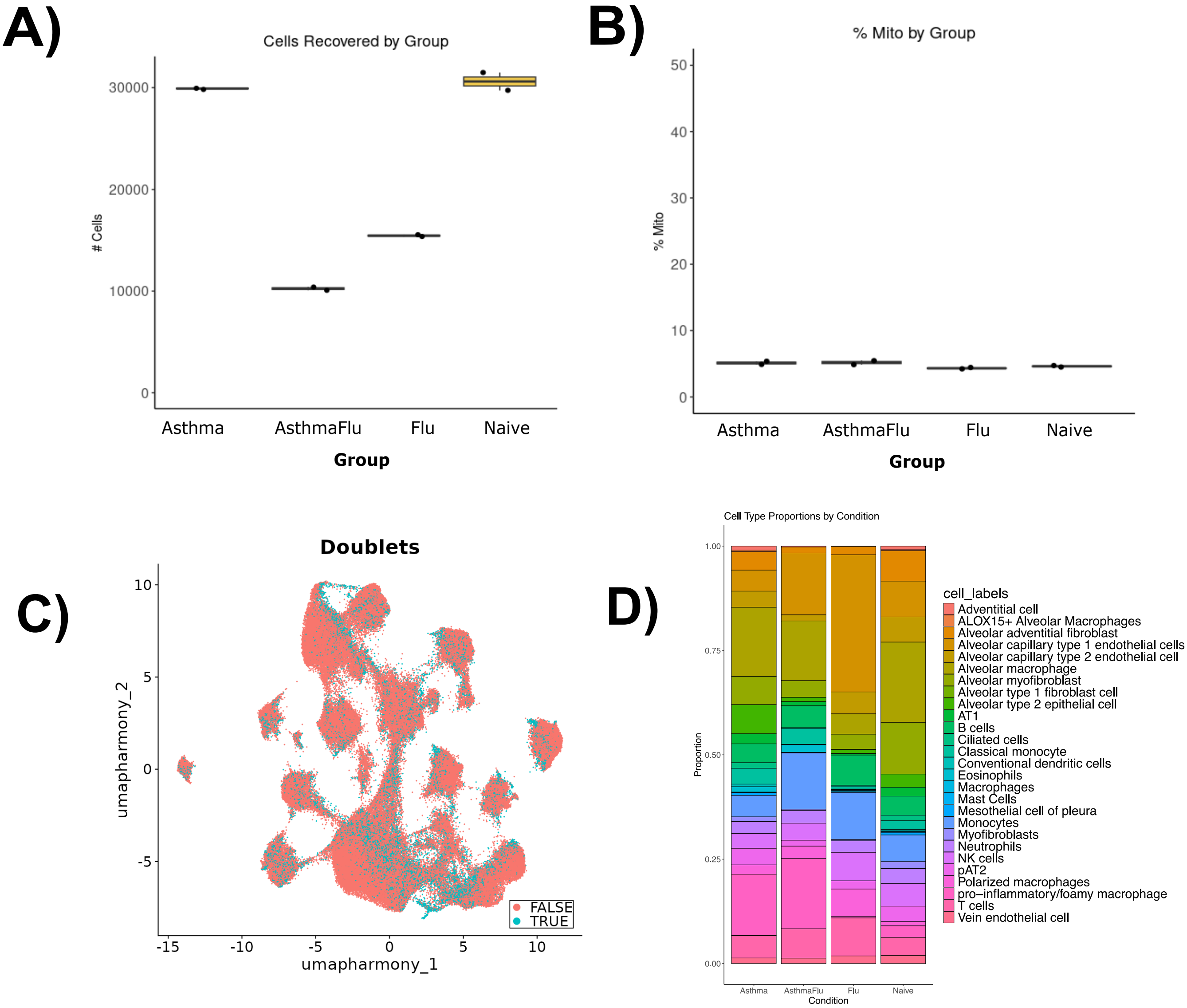

Supplementary Figure 1. Quality control and metrics associated with scRNA-seq
A) Number of cells recovered per group. B) Percent mitochondrial RNA per group. C) Detected doublets before removal from downstream analysis. D) Stacked bar plots of cell type compositions for all groups.

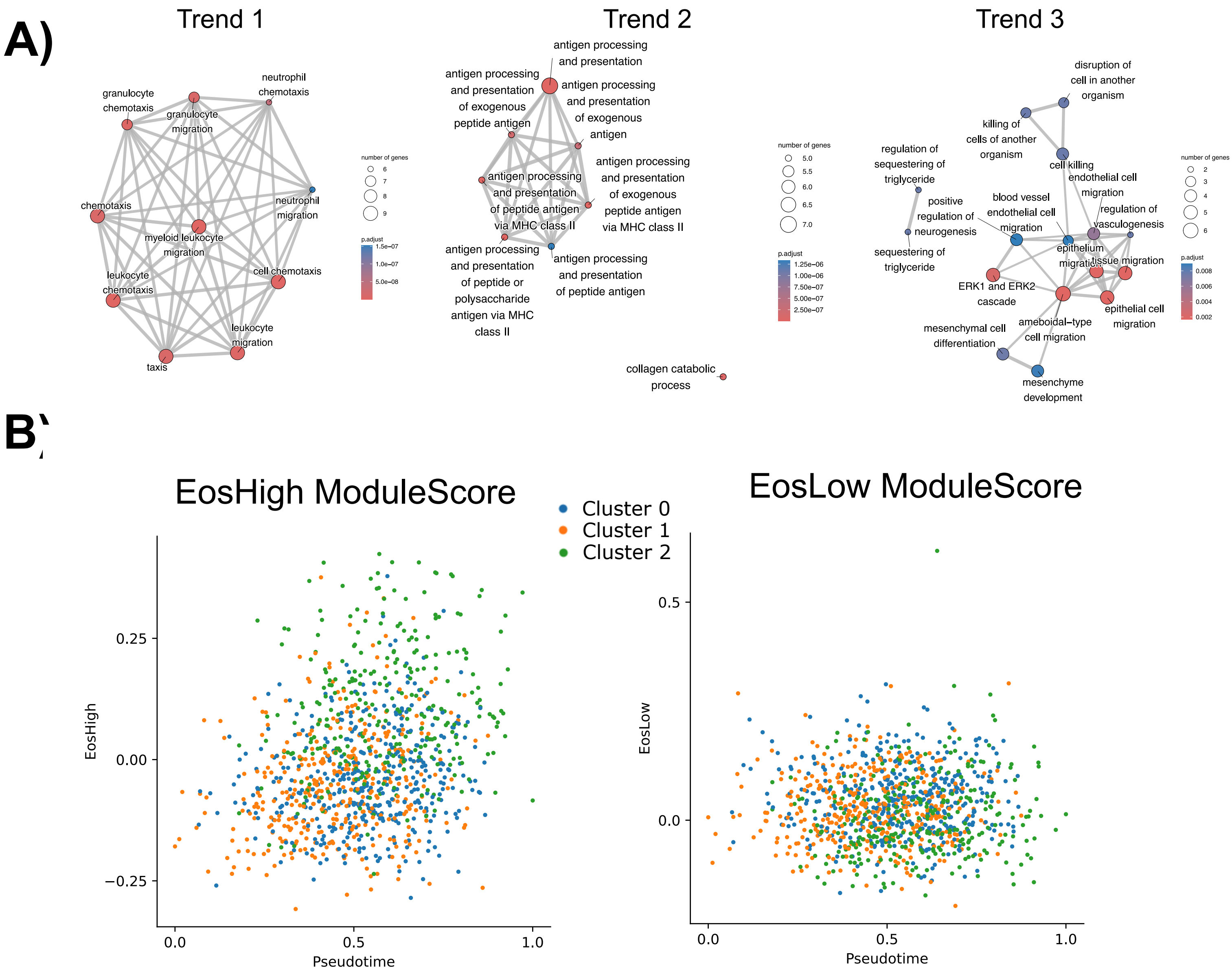

Supplementary Figure 2. Pathways and gene signatures associated with eosinophils by pseudotime analysis
A) Enriched GO pathways associated with each gene trend represented as network plots (emapplot). B) EosHigh and EosLow gene signatures from Doni Jayavelu, et al. [14] as ModuleScores scores plotted vs. pseudotime.

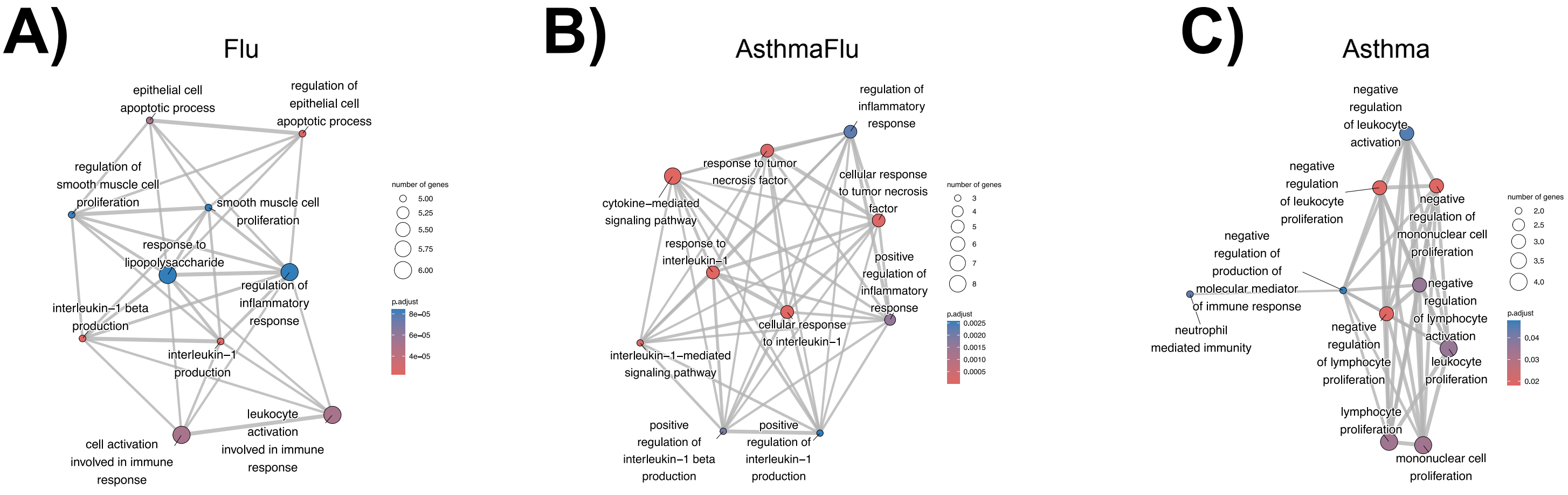

Supplementary Figure 3. Pathways associated with mast cells by condition

Enriched GO pathways associated with each gene trend represented as network plots (emapplot) for A) Flu vs. Naïve comparison B) Asthma+Flu vs. Naïve comparison C) Asthma vs. Naïve comparison.

A)

condition	sample	n_fov	n_cells
Naive	11	29	12927
Naive	13	44	22347
Naive	15	34	15461
Asthma	1	125	107394
Asthma	2	105	85337
Asthma	3	131	111752
AsthmaFlu	10	82	63966
AsthmaFlu	7	105	90457
AsthmaFlu	8	115	83660
Flu	17	80	55735
Flu	19	109	83493
Flu	20	92	83052

B)

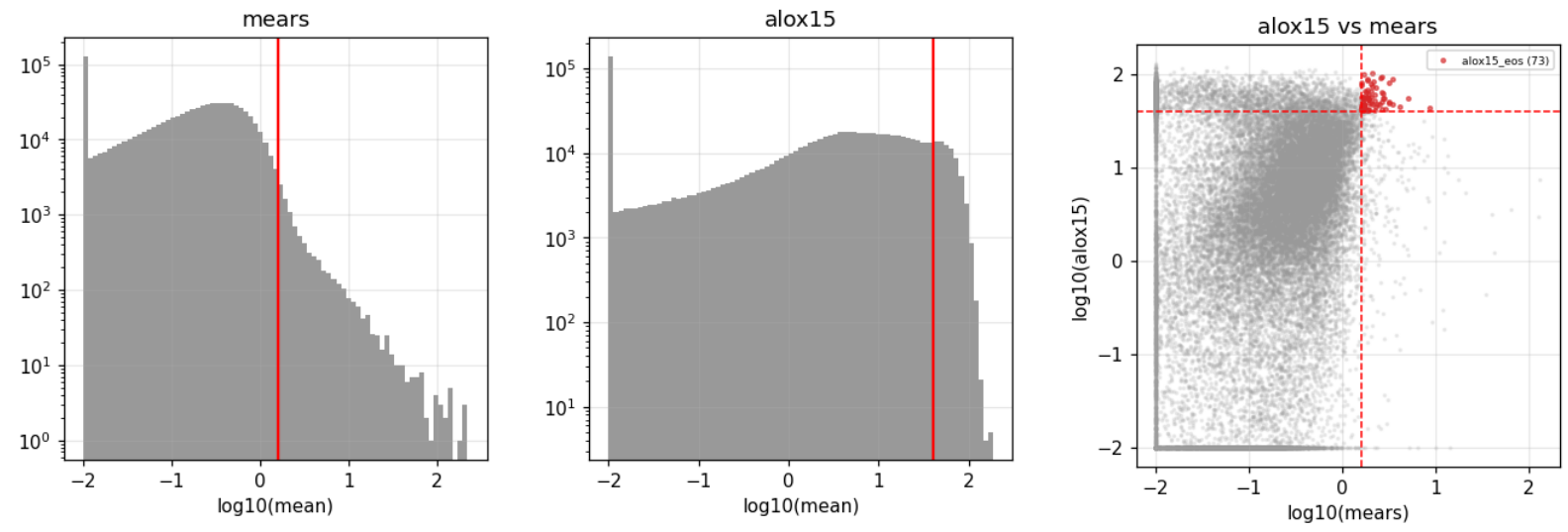

Supplementary Figure 4. Statistics and thresholding for immunofluorescence analysis
A) A table showing the number of fields of view (FOVs) for each sample and the number of cells detected by nuclear segmentation & expansion with StarDist. B) Histograms and scatter plot of ALOX15 and mEARs signal quantified within cell segmentation boundaries. The vertical red line in histograms and dotted lines in scatter plot indicate chosen thresholds above which cells were considered positive for the marker. Cells with no detectable expression included in histograms using a pseudocount of 0.01 (-2 on log10 scale).

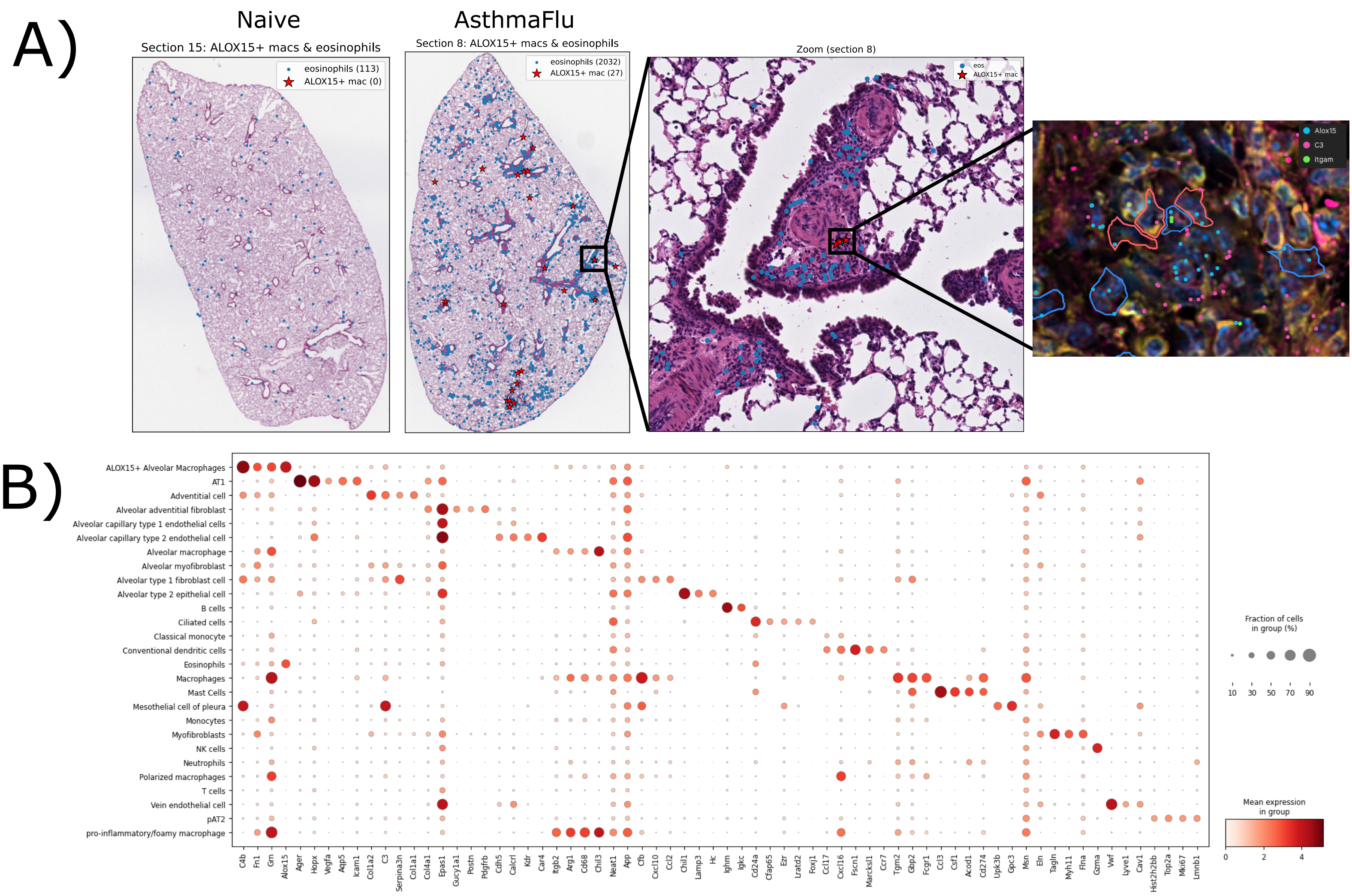

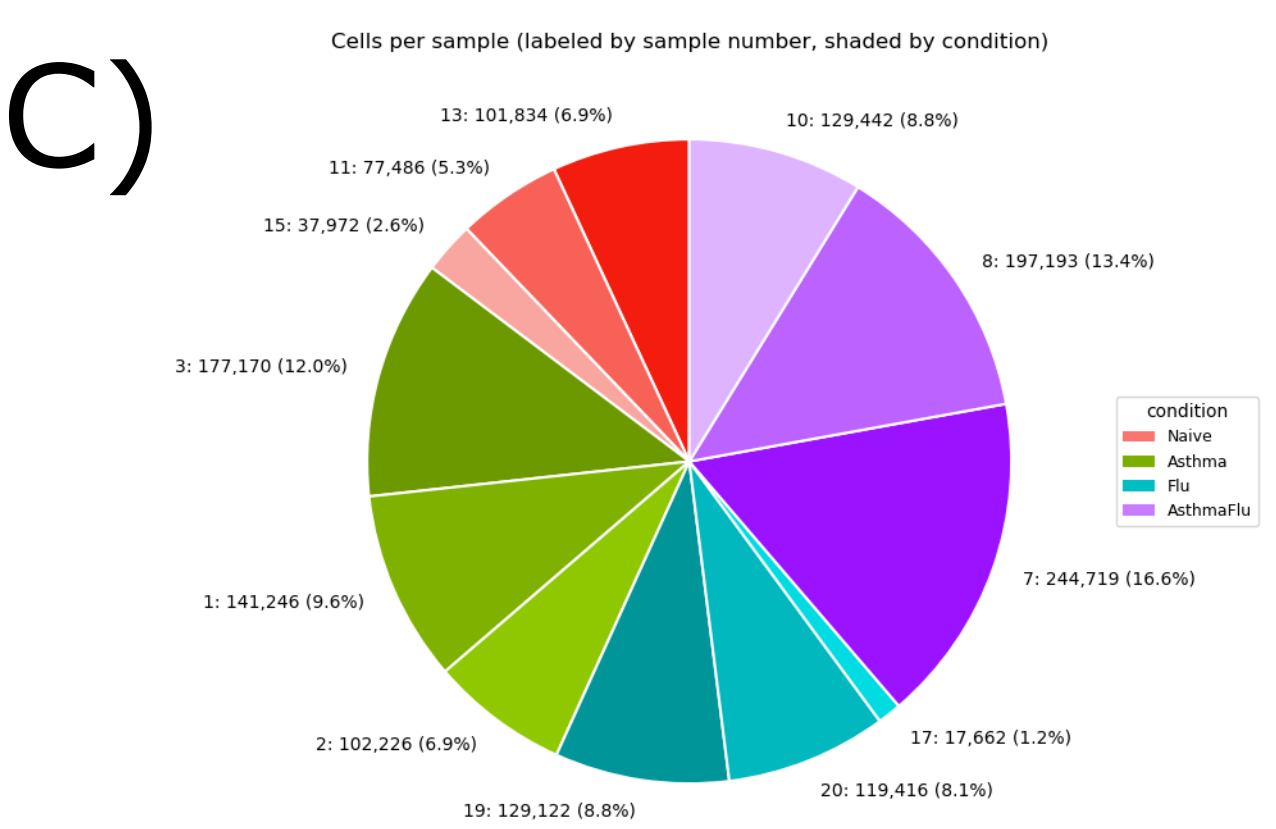

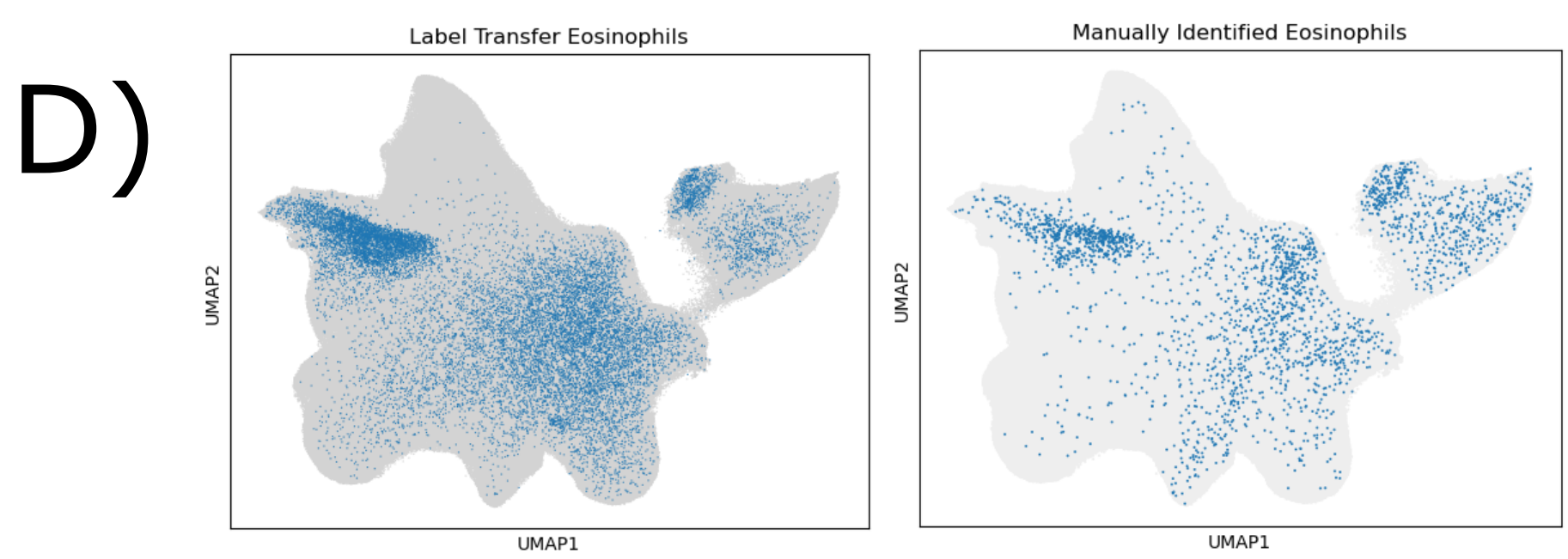

condition	Eosinophils n	Eosinophils %	ALOX15+ Alveolar Macrophages n	ALOX15+ Alveolar Macrophages %	Mast Cells n	Mast Cells %
Naive	557	0.256	13	0.006	0	0.000
Asthma	7326	1.742	111	0.026	4	0.001
Flu	3443	1.293	28	0.011	15	0.006
AsthmaFlu	4589	0.803	57	0.010	8	0.001

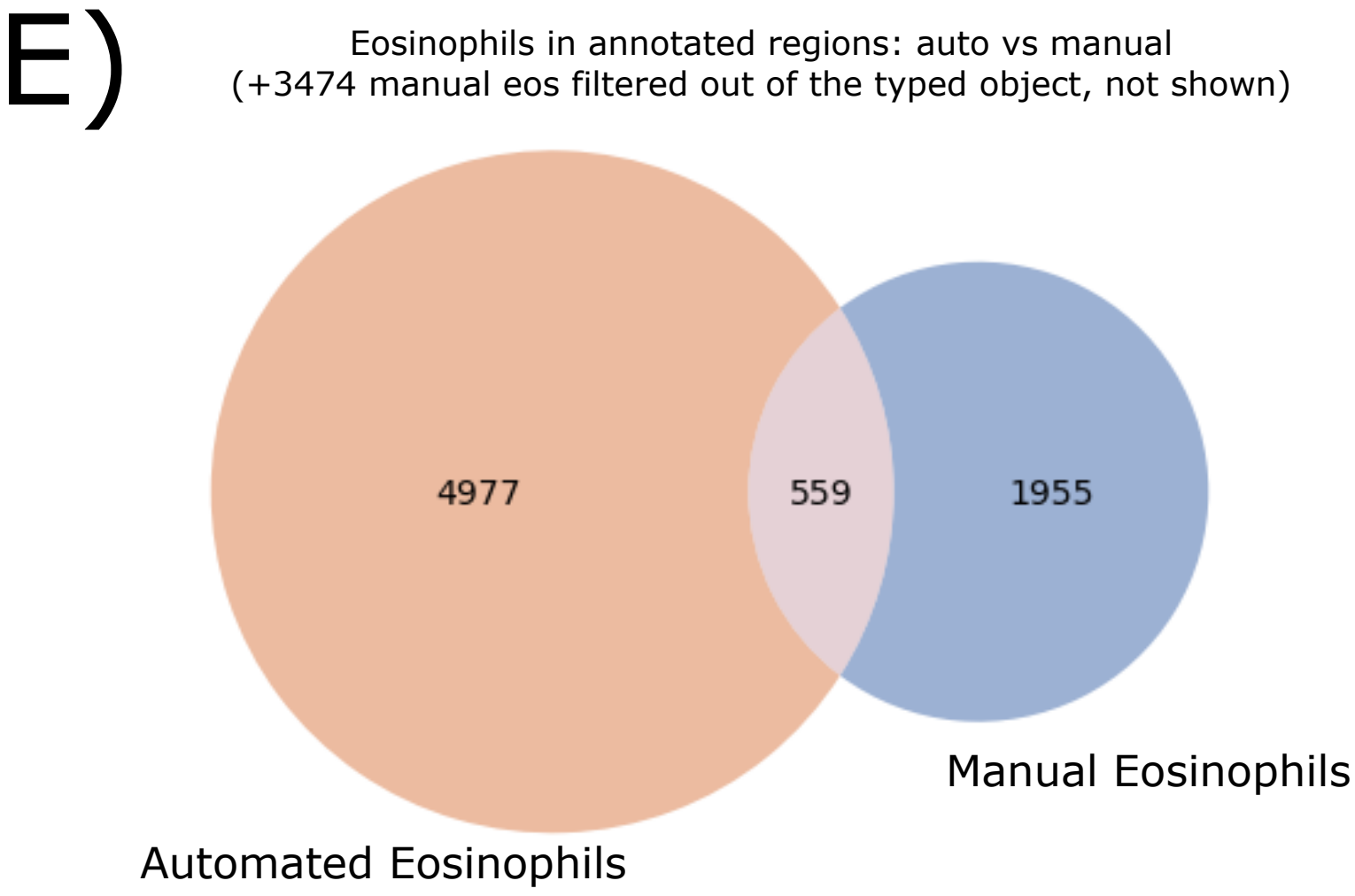

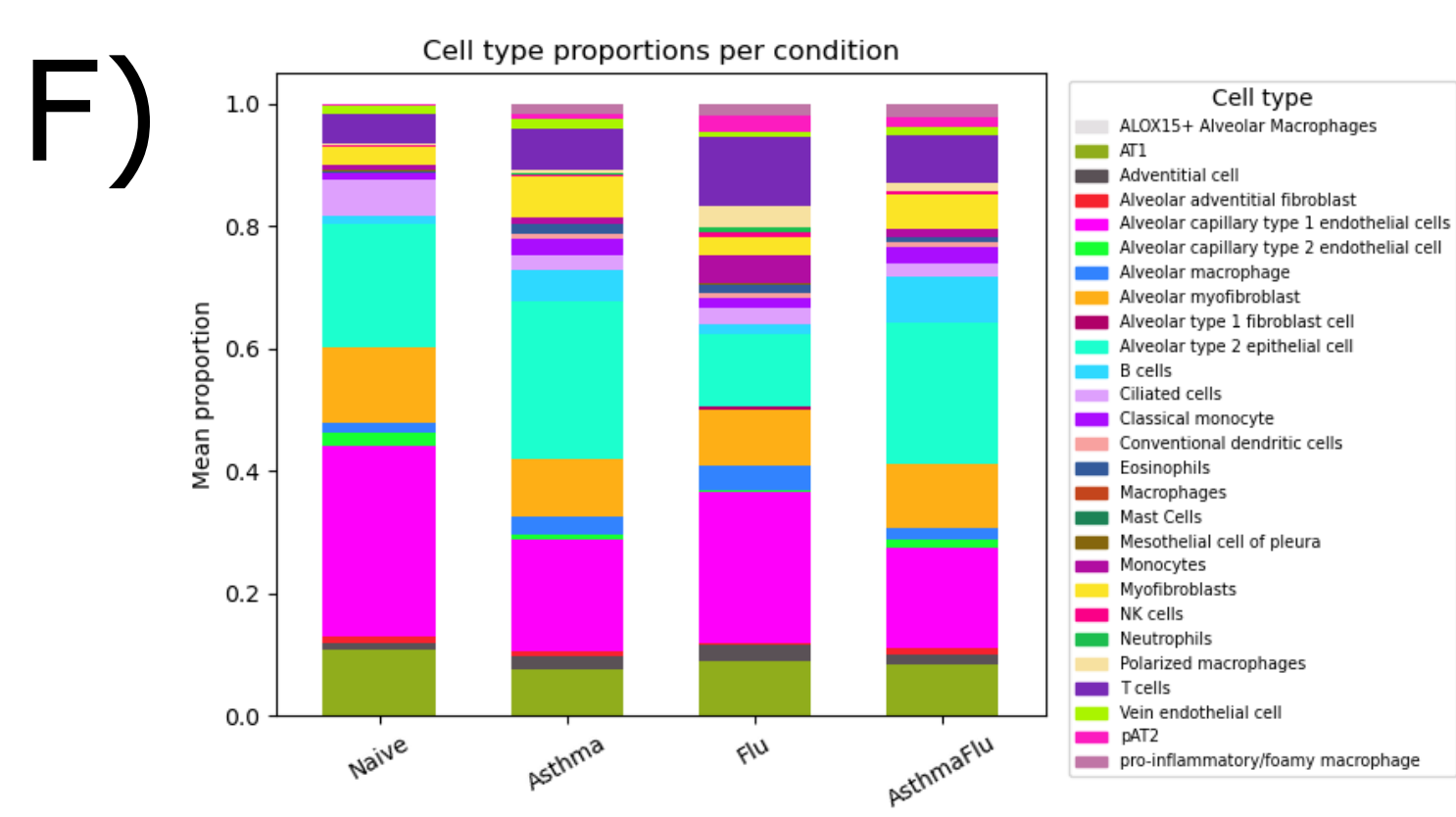

Supplementary Figure 5. Xenium Spatial transcriptomics QC, cell type prediction and eosinophil identification
A) Selected images showing Xenium spatial transcriptomics. Aligned H&E stains are shown for a selected Naïve (sample 15) and Asthma+Flu (sample 8) tissue section. Predicted eosinophil (blue circles) and Alox15+ macrophages (red stars) are marked. Three successive zoom levels are shown for sample 8, highlighting an area where Alox15+ macrophages and eosinophils are colocalized. The final zoom, right, shows Xenium marker fluorescence and the locations of Alox15, C3, and Itgam, genes we identify as important for the interactions of these cell types. B) Dot plot showing top marker gene expression for predicted cell types from Xenium. Marker genes defined as >30% expression. C) Pie chart showing contributions from each sample, colored by disease condition. D) Top, UMAP annotation of CellTypist automated eosinophil annotations, compared with manually identified eosinophils from selected regions. Distributions are largely similar. Bottom, counts and percentages of automated identification of eosinophils, Alox15+ macrophages and mast cells among xenium cells with >28 UMIs detected. E) Venn diagram of overlap between the manually identified and automated identification of eosinophils within selected regions. F) Stacked bar charts showing the proportions of each cell type in each disease condition.

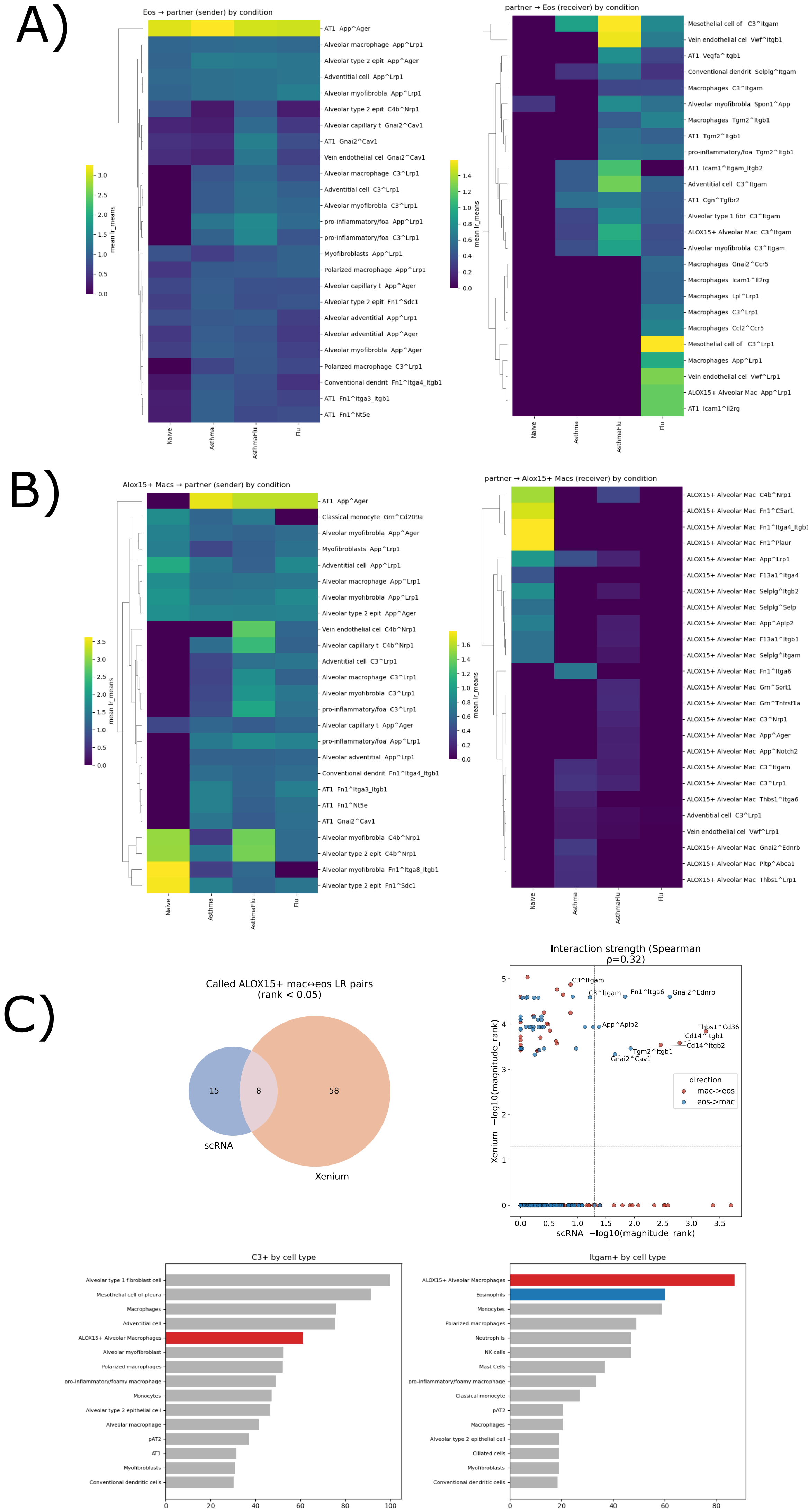

Supplementary Figure 6. LIANA+ cell cell communication analysis and comparison with scRNA-seq
A) Left, top interactions averaged by condition with eosinophils as senders. Right, top interactions averaged by condition with eosinophils as receivers. B) Left, top interactions averaged by condition with Alox15+ macrophages as senders. Right, top interactions averaged by condition with Alox15+ macrophages as receivers. C) Top left, a pie chart showing which interactions from LIANA analysis of scRNA-seq data and LIANA analysis of spatial transcriptomics data achieved magnitude_rank < 0.05. Top right, magnitude rank from scRNA-seq and spatial transcriptomics for shared pathways. Dotted lines indicate 0.05 magnitude rank. Top shared pathways are labeled. Bottom, C3 and Itgam gene expression positivity rate from scRNA-seq, showing which cell types have the most expression abundance.
